# Cold-responsive miRNAs and their target genes in the wild eggplant species *Solanum aculeatissimum*

**DOI:** 10.1186/s12864-017-4341-y

**Published:** 2017-12-29

**Authors:** Xu Yang, Fei Liu, Yu Zhang, Lu Wang, Yu-fu Cheng

**Affiliations:** College of Horticulture and Plant Protection, Yangzhou University, Yangzhou, 225009 China

**Keywords:** Cold stress, *Solanum aculeatissimum*, High-throughput sequencing, qRT-PCR

## Abstract

**Background:**

Low temperature is an important abiotic stress in plant growth and development, especially for thermophilic plants. Eggplants are thermophilic vegetables, although the molecular mechanism of their response to cold stress remains to be elucidated. MicroRNAs (miRNAs) are a class of endogenous small non-coding RNAs that play an essential role during plant development and stress responses. Although the role of many plant miRNAs in facilitating chilling tolerance has been verified, little is known about the mechanisms of eggplant chilling tolerance.

**Results:**

Here, we used high-throughput sequencing to extract the miRNA and target genes expression profiles of *Solanum aculeatissimum* (*S. aculeatissimum*) under low temperature stress at different time periods(0 h, 2 h, 6 h, 12 h, 24 h). Differentially regulated miRNAs and their target genes were analyzed by comparing the small RNA (sRNA) and miRBase 20.0 databases using BLAST or BOWTIE, respectively. Fifty-six down-regulated miRNAs and 28 up-regulated miRNAs corresponding to 220 up-regulated mRNAs and 94 down-regulated mRNAs, respectively, were identified in *S. aculeatissimum*. Nine significant differentially expressed miRNAs and twelve mRNAs were identified by quantitative Real-time PCR and association analysis, and analyzed for their GO function enrichment and KEGG pathway association.

**Conclusions:**

In summary, numerous conserved and novel miRNAs involved in the chilling response were identified using high-throughput sequencing, which provides a theoretical basis for the further study of low temperature stress-related miRNAs and the regulation of cold-tolerance mechanisms of eggplant at the miRNA level.

**Electronic supplementary material:**

The online version of this article (10.1186/s12864-017-4341-y) contains supplementary material, which is available to authorized users.

## The study design

In this paper, low temperature treatment of cold-resistant eggplant *Solanum aculeatissimum* was carried out, and cold-resistant miRNAs of eggplant were excised by transcriptome and small RNA sequencing. We used high-throughput sequencing to extract the miRNA and target genes expression profiles of *S. aculeatissimum* under low temperature stress at different time periods(0 h, 2 h, 6 h, 12 h, 24 h). First, the small RNA library was established, and the expression of miRNAs and target genes was calculated by RPKM and Expdiff, that searched the differential expression of miRNAs and its target genes. By analyzing the enrichment of GO and KEGG, the cold tolerance related genes were analyzed, and the mechanism of cold tolerance related miRNAs in eggplant was explored.

## Background

In nature, the growth of plants is inseparable from temperature, which is one of the factors affecting their geographical distribution. Cold tolerance is different for every plant as well as among varieties of the same plant. Under low-temperature stress, plants with strong cold tolerance experience less temperature-related damage than plants with weaker cold tolerance, and the duration of their low-temperature tolerance is greater. To overcome cold stress, plants have modified their own cold tolerance by evolving various adaptive mechanisms, which lead to changes in gene expression and subsequent physiological enhancement. These physiological responses include induction of transient increases in Ca^2+^ and ABA levels, alterations in lipid composition, increases in antioxidant levels, and the accumulation of osmoprotectants [1].

MicRNAs (miRNAs) are endogenous, non-coding, single-stranded small RNA (sRNA) that are approximately 22 nucleotides in length, and are important regulators of plant growth and development. miRNAs promote the degradation and inhibit translation of target genes by binding to the 3′ untranslated region, so that post-transcriptional gene expression levels are regulated in different biological and metabolic processes. Plant miRNAs are involved in a range of activities, including adverse environmental responses to temperature, drought, salt, nutrient starvation, heavy metal stress [2–5], and defensive responses to pathogenic infection [6]. miRNAs that respond to low temperature stress are divided into three categories according to their target gene types. The first category targets genes that directly respond to stress and external stimuli, the second category targets genes that indirectly respond to stress by regulating protein transcription factors that play a role in stress responses, and the third category is made up of miRNAs that can respond to multiple stresses and that target genes coding for either hydrolases or oxidoreductases [7]. The C-repeat binding factor (CBF)/dehydration responsive element provides one of the most important pathways for cold response for a large number of cold regulatory genes that encode transcription factors or proteins involved in transcription. It has been shown in transgenic eggplants that heterologous expression of Arabidopsis C-repeat binding factor 3(*AtCBF3*) and cold-regulated 15A (*AtCOR15A*) enhanced chilling tolerance [1, 8].

Currently, many international studies focus on the role of miRNAs in abiotic stress in various plants. Some miRNAs related to low-temperature stress in plants have been studied and verified, and the regulatory mechanisms of plant responses to low-temperature stress is also well understood [9]. Many studies have been carried out using Solanaceae plants [10–18], however, few studies focus on the miRNAs of eggplant, especially wild eggplant species.

Eggplant is one of the world’s top 10 vegetable crops and has multiple different varieties and growth habits. Among the three types of Solanaceae (tomatoes, peppers and eggplants) tomatoes have the strongest cold tolerance, followed by peppers, and the cold tolerance of eggplants is the lowest. The growth of eggplants below 15 °C was slow and caused the flower to fall. In addition, the metabolism was lower at 10 °C. In this huge family, there is no lack of cold-resistant characteristics found in different eggplant varieties. The cold resistance of the wild eggplant is very strong and therefore worth studying. *Solanum aculeatissimum* (*S. aculeatissimum*) is one species of wild eggplant and is also known as bitter bell eggplant, thorn eggplant or barbed eggplant. It originated in Brazil, where it was originally grown in the mountain forest and at the edge of fields among weeds. It is now widely distributed in tropical Asia and Africa as well. In China, *S. aculeatissimum* is mainly distributed in Yunnan, Guizhou and other locations. It is not found in Jiangsu and the surrounding areas, but it is found in India, usually in roadside shrubs, wastelands, grassy slopes, and open forests, at elevations of 1300–2300 m above sea leve [19]. There are a number of related wild species; however, most cultivated eggplant species lack important medicinal properties when in the form of fresh or dried fruits and leaves. Previous results showed that the fruit of *S. aculeatissimum* contains solasodine, which is an important precursor of synthetic steroids and sex hormones, and has detoxification effects [20–22]. Therefore, the adaptability and cold tolerance of *S. aculeatissimum* provide a good basis for study of the mechanism of cold tolerance in eggplants and are useful references for cultivating cold-tolerant variants.

To elucidate the mechanisms of miRNA regulation in *S. aculeatissimum*, we used the transcriptome and small RNA sequenceing to *S. aculeatissimum* under low-temperature stress, and analyzed the correlation between mRNA and miRNA, which will help to clarify the responses of miRNAs and their target genes in eggplants at low temperature and the miRNA-regulated mechanism of cold tolerance in eggplant plants.

## Methods

### Plant materials and treatments

The wild eggplant cultivar *S. aculeatissimum* was obtained from the eggplant molecular breeding laboratory at Yangzhou University. Seeds were soaked in 55 °C water and gauze wrapped in a climate chamber (specifications) with an air temperature of 25 °C for germination. After seeding, seedlings were placed into a controlled environment of 20–30 °C, 75% humidity and 15,000–20,000 lx, until the seedlings grew into 3–4 true leaves. The growth potential of the same seedlings was analyzed at low temperature (1 °C). Environmental processing and other environmental parameters remained the same. The test materials were divided into control group and treatment group. The control group was that the seedlings grow at normal temperatures (0 h). The treatment group was that the seedlings grow at low temperature environment (1 °C) for 2, 6, 12 and 24 h. The third fully expanded leaf was collected at o h, 2 h, 6 h, 12 h and 24 h after treatment, immediately frozen in liquid nitrogen and stored at −80 °C. RNA samples were extracted from five cold treatment time and stored at −80 °C. All of RNA samples taken at 0 h, 2 h, 6 h, 12 h and 24 h cold treatment were subjected to transcriptome and small RNA sequencing by high-throughput sequencing (Berry Genomics Corporation, Beijing, China). The samples taken at 0 h, 2 h, 6 h, 12 h and 24 h also used for qRT-PCR analysis.

### RNA preparation for high-throughput sequencing

Total RNA of each sample was extracted using the Takara, The integrity of the RNA samples was assessed with 1% agarose gel electrophoresis and RNA extract was stored at −80 °C.

After extracting the total RNA and digesting the DNA with DNase I, the eukaryotic mRNA was enriched with a magnetic beads bearing Oligo (dT); the interruption reagent was added to the thermomixer to interrupt the mRNA into short fragments to interrupt. And then, the two-stranded cDNA was synthesized using a two-stranded synthesis system. The two-stranded cDNA was synthesized and purified using a kit. The end of the cDNA was repaired, the 3 ‘end of the cDNA was added with the base “A” and the linker was ligated. The size of the fragment was selected and the PCR amplification was carried out. Finally, the constructed library was sequenced using the Agilent 2100 Bioanalyzer and ABI StepOnePlus Real-Time PCR System, followed by Illumina HiSeq.

### Construction and sequencing of sRNA library

The total RNA was extracted and the RNA of different fragment size was separated using PAGE, then cut the band between 18 and 30 nt to recover sRNA; 5′ joint connection system was prepared after mixing and centrifugation at the appropriate temperature for a certain time, and the reaction product was prepared for mixing 3′ linker at the same way, then replace the 3′ ligation product with PAGE. The reverse transcription system was prepared and reacted at a suitable time on the PCR instrument for a certain time, that the linked product is reverse transcribed, the PCR products were digested and purified. Agilent 2100 Bioanalyzer and ABI StepOnePlus Real-Time PCR System for quality and yield detection. HiSeq obtained 49 nt sequence, after the filter to obtain a trusted target sequence, we figured out the quality and length of these sequences, besides the common sequence between the samples. Through the target sequence classification notes, we could get the information of the components and expression. The prediction of unknown miRNAs was conducive to the clustering analysis and target gene prediction of known and novel miRNAs, besides to GO function and KEGG pathway annotation of target genes.

### Raw sequence processing and de novo assembly

Sequencing reads were not all valid, which contains the node, the repetition of the sequencing in the very low quality of the reads, these reads would affect the assembly and follow-up analysis, we filtered the machine’s reads, removed the adaptor read, the N ratio of more than 5% of the reads, and low quality reads (the base number of the mass value of Q ≤ 10 accounts for more than 20% of the entire read). Such clean reads were obtained.

We used short reads to assemble the software Trinity, the result of what we called Unigene. First used Tgicl to deallocate and further stitch, and then homologous transcripts of these sequences to get the final Unigene.

The Unigene sequence was compared with the protein database NR, Swiss-Prot, KEGG and COG for blastx (evalue <0.00001), and the sequence of Unigene was determined by comparing the best results. If there is a contradiction between the results of the comparison of the different libraries, the order of the sequence of Unigene is determined according to the priority of NR, Swiss-Prot, KEGG and COG. Unigene is not comparable to the above four libraries. We used the software ESTScan to determine the sequence direction.

### Data analysis of sRNA and identification of conserved and novel miRNAs

Expression levels of miRNAs were determined based on sequence analysis of mature miRNAs that share the same sequence and expression levels. After sequencing, raw reads were obtained using Illumina sequencing-related software analysis. Next, raw reads with low quality and low copy numbers were removed. We obtained sequences with lengths between 19 and 24 nucleotides that were subjected to analysis using miRBase 20.0 (http://www.mirbase.org/) for conserved and novel miRNA identification.

### Expression difference statistical analysis

FPKM (fragments per kb per million fragments) was used to calculate unigene expression levels. The FDR (False Discovery Rate) control method was applied to determine the threshold of *p* value in multiple tests and analyses. After the FDR was obtained, the ratio of FPKM was used to calculate the fold-change in the expression of unigenes in two samples simultaneously. An FDR < 0.001 and the absolute value of log2-ratio ≥ 1 were used as the threshold for the judgment of the significance of the gene expression differences. The different expressed genes were then subjected to GO and KEGG ontology enrichment analysis. Comparing the known or novel miRNAs expression between two samples to find out the differentially expressed miRNAs by Expdiff. First, normalized the expression of miRNAs in two samples (control and treatment) to get the expression of transcript per million (TPM). Second, calculated fold-change and *p* value. Last, generated the log2-ratio and scatter plot. That the expression of miRNAs was showed in two samples by plotting log2-ratio and scatter plot, which was found to be significantly up-regulated or down-regulated when the chi-square test and Fisher-exact test resulted in *p* ≤ 0.05 and |log2| ≥ 1, besides, miRNAs with different expression levels were used for GO enrichment analysis.

### Correlation analysis of sRNA and target gene mRNA

To analyze the modulatory effects of chilling stress on miRNA expression, we compared the distribution of differentially expressed miRNAs between control and chilling stress treatments over different time periods. The principle of screening cold-tolerant miRNAs and their target genes is that they occur at least simultaneously in two groups of differentially expressed, means that miRNAs differential expression in at least two groups of comparison at 0-2 h, 0-6 h, 0-12 h, 0-24 h, 2-6 h, 6-12 h and 12-24 h; the same time target genes must also at least two groups of differentially expressed at 0-2 h, 0-6 h, 0-12 h, 0-24 h, 2-6 h, 6-12 h and 12-24 h, that is the cold tolerance candidate miRNA and target genes. Finally, we obtained miRNAs with differentially down-regulated and up-regulated miRNAs with their corresponding up-regulated and down-regulated target genes. We further screened for miRNAs that differed under low temperature stress using significant binding assays, which correlate to sRNAs and target genes.

### Functional analysis of target genes

To elucidate the function of target genes, GO function annotation and KEGG pathway analysis were performed using the GO database (http://www.geneontology.org/) and KEGG database (http://www.genome.jp/kegg/), respectively. Sequencing of miRNAs based on sRNA assembly was used to determine differential expression levels and degradation of their target genes. The function of target genes was determined according to the GO function.

### Analysis of miRNAs by qRT-PCR

SRNAs were extracted from *S. aculeatissimum* seedlings exposed to cold treatment or no cold treatment for 0, 2, 6, 12 and 24 h using RNAiso.

The miRNAs were detected by stem-loop RT-PCR using the following primer 5′-GTTGGCTCTGGTGCAGGGTCCGAGGTATTCGCACCAGAGCCAAC-3′. Primers for target genes were designed using Primer 5.0. qRT-PCR was performed using the primers in Additional file table S4. qRT-PCR was performed on the ABI 7500 Real-time PCR system using a SYBR Green-based PCR assay and qRT-PCR samples contained 2 μL specific reverse transcription cDNA, 10 μL SYBR Green (Takara), 1 μL universal primer, 1 μL miRNA-specific reverse transcription primer, supplemented with water to 20 μL. qRT-PCR reactions of miRNAs and target genes were performed as follows: 95 °C for 5 min, followed by 40 cycles of 95 °C for 5 s and Tm for 30 s. The expression levels were calculated using the 2^−ΔΔCt^ method. qRT-PCR data were analyzed by SPSS 16.0.

## Results

### De novo assembly

The *S. aculeatissimum* seedlings were divided into five time periods under low temperature(0 h, 2 h, 6 h, 12 h and 24 h), which were groups of total RNA. A total of 425,099,682 raw reads were generated for five time periods, after discarding the low-quality reads, a total of 425,087,254 (total clean nucleotides 53,135,906,750 nt) clean reads were obtained. Besides, the Q20 percentage was over 96%. The short reads were assembled into 180,292 contigs, and the average contig size was 384 nt with the N50 of 736. The contigs were further assembled into unigenes, generating 95,642 unigenes with a mean length of 1028 bp (Table 1).

**Table 1:** Summary for the *S. aculeatissimum* transcriptome

	Total
Total Raw Reads	425,099,682
Total Clean Reads	425,087,254
Total Clean Nucleotides (nt)	53,135,906,750
Q20 percentage	96.63%
N percentage	0.00%
GC percentage	44.21%
Contig	
Total Number	180,292
Total Length (nt)	69,159,499
Mean Length (nt)	384
N50	736
Unigene	
Total Number	95,642
Total Length (nt)	98,283,273
Mean Length (nt)	1028
N50	1804
Total Consensus Sequences	95,642
Distinct Clusters	34,158
Distinct Singletons	61,484

### Functional annotation and classification

All of the unigenes were tested with BLAST analysis against protein databases with a cutoff e value of 10^−5^. The public protein databases included the NCBI non-redundant protein(Nr) database, the Clusters of Orthologous Groups (COG)databases, the Swiss-Prot protein database, and the KyotoEncyclopedia of Genes and Genomes (KEGG) database. 54,736, 70,274, 32,938, 31,582 and 19,987 unigenes were found in NR, NT, Swiss-Prot, KEGG and COG, respectively.

**Fig. 1:**
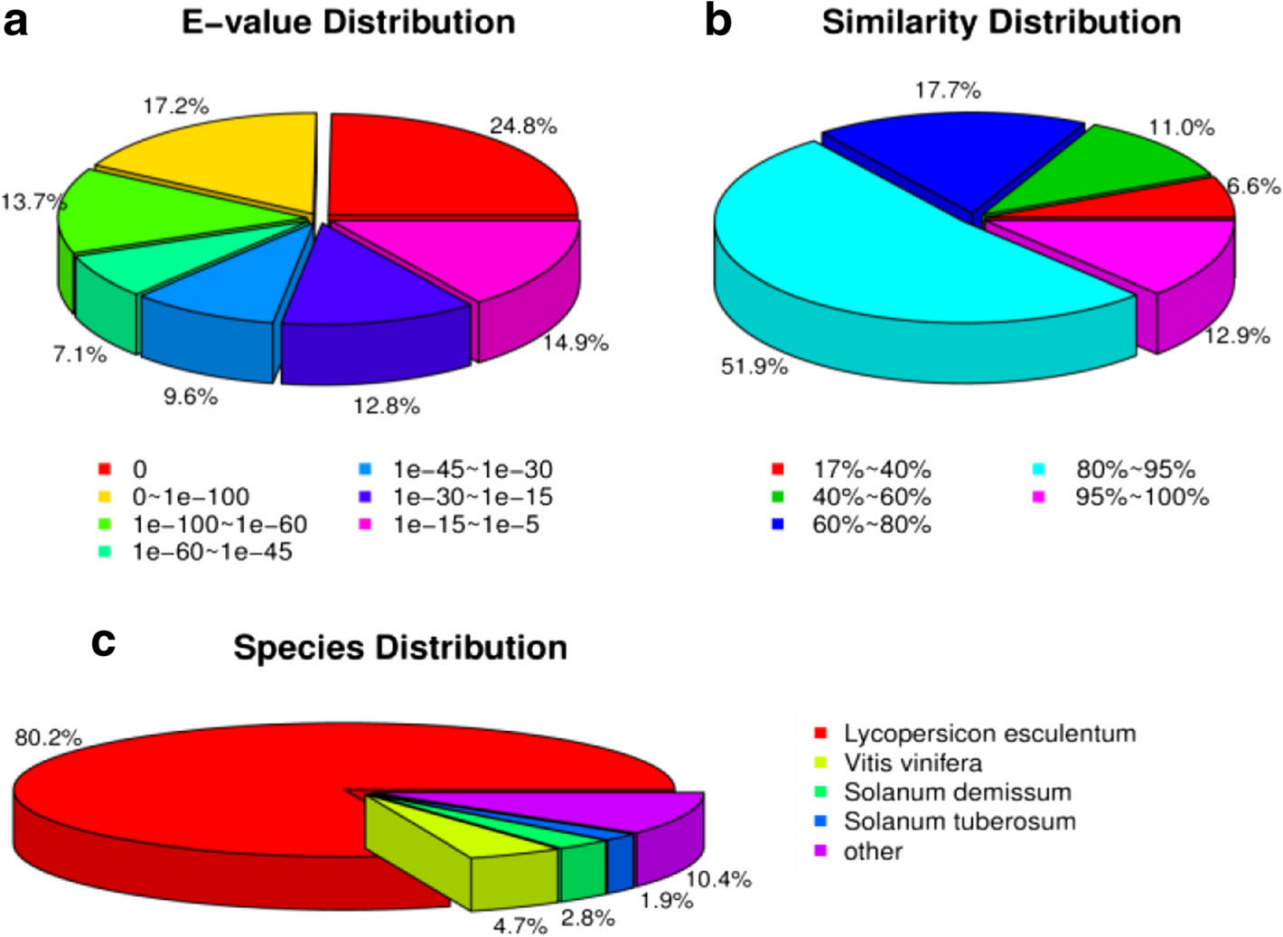
NR annotation of Unigene. **a** The e-value distribution of the NR annotation; (**b**) the similarity distribution of the NR annotation; (**c**) the distribution of the NR annotations

The Unigene sequence was aligned to the protein database NR (evalue <0.00001) by blastx, giving a Unigene database comment to NR and classification information (Fig. 1).

**Fig. 2:**
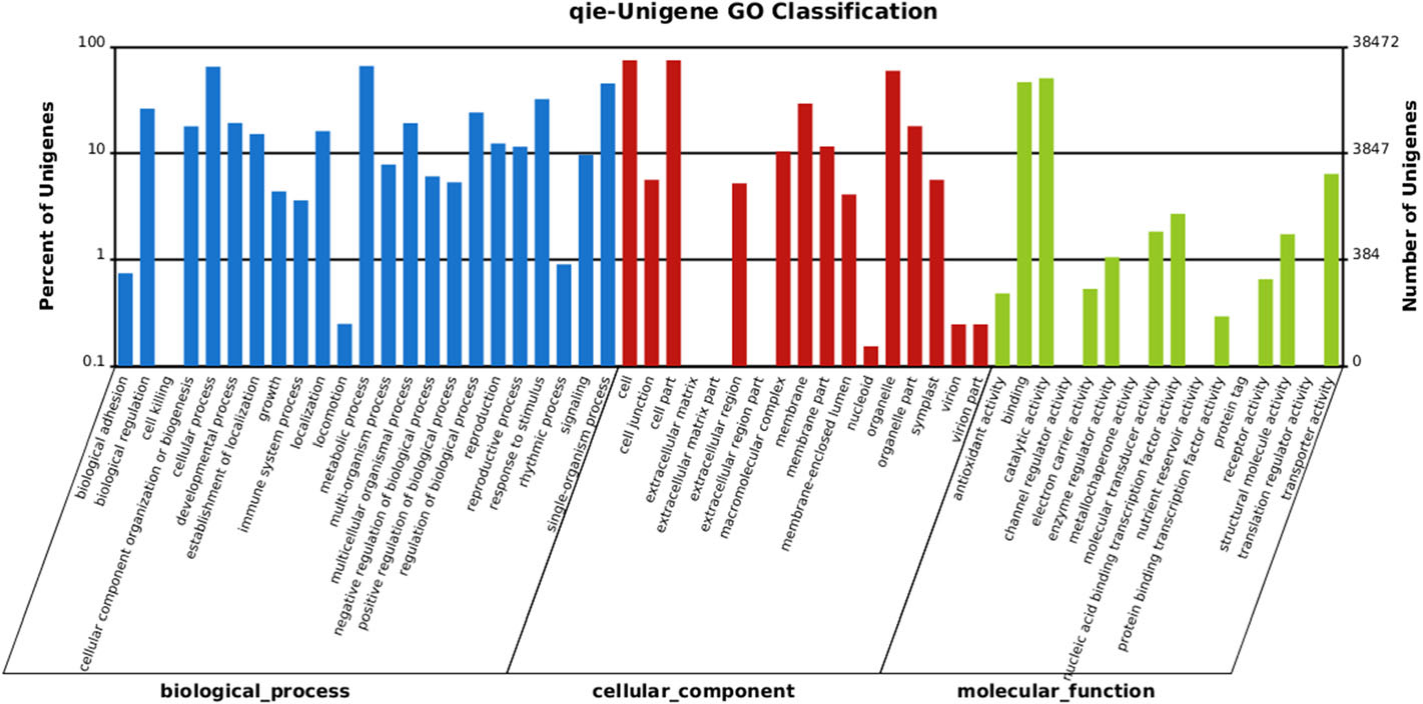
GO annotation of Unigene. The X-axis is the category of the GO function. The Y-axis on the right indicates the number of Unigene that is commented to the corresponding GO function. The Y-axis on the left indicates the percentage of the total number of Unigene

The GO classification system was used to classify the possible functions of the unigenes based on Nr annotations. A total of 38,472 unigenes were classified into three main categories, including biological process(BP), cellular component(CC) and molecular function(MF), which were then categorized into 56 functional groups. For biological process category, the top five largest categories were: “metabolic process” (25387), “cellular process” (24973), “single-organism process” (17389), “response to stimulus” (12492), and “biological regulation” (10157). For cellular components, the top three categories were: “cell” (28727), “cell part” (28727), and “organelle” (22882). For molecular function, the top three categorieswere: “catalytic activity” (19476), “binding” (17884), and “transporter activity” (2463) (Fig. 2).

**Fig. 3:**
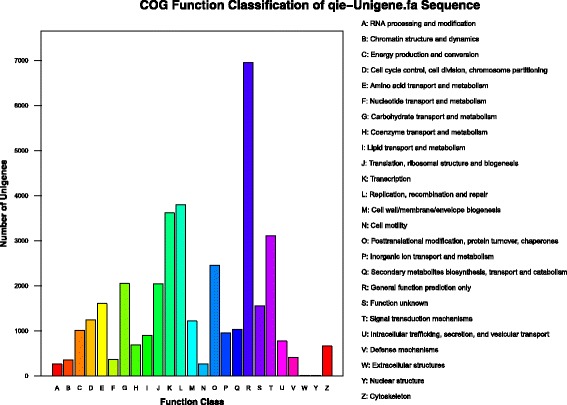
COG annotation of Unigene. The abscissa is the classification number of the COG function, and the ordinate is the number of Unigene corresponding to the classification. Right side of the figure is the full name of the COG function classification number

The all-unigenes were aligned to the COG database in which orthologous gene products were classified. A total of 19,987 genes were identified and assigned into 25 functional clusters. Among these COG categories, the cluster “general function prediction only” was the largest group (6954 of 19,987 unigenes, 34.8%), followed by “replication, recombination and repair” (3799 of 37,340 unigenes, 18.9%), “transcription” (3616 of 37,340 unigenes, 18.1%), “Signal transduction mechanisms” (3109 of 37,340 unigenes, 15.5%) (Fig. 3).

The BLAST analysis of all-unigenes was also performedagainst the KEGG database to further demonstrate functional classification and pathway assignment. In total, 31,582 unigenes were involved in 128 KEGG pathways. The major pathways were “metabolic pathways,” with 6796 of 31,582 unigenes (21.52%) related to it, followed by “biosynthesis of secondary metabolites” with 3466 unigenes (10.97%) involved, “plant-pathogen interaction” with 1935 unigenes(6.13%) involved, “plant hormone signal transduction” with 1869 unigenes (5.92%) involved, and “RNA transport” with1837 unigenes (5.82%) involved.

### Protein-coding region prediction

A total of 54,424 coding sequences (CDS) from unigene sequences were extracted and translated into peptide sequences after subjecting all-unigene sequences to BLASTx (e value < 0.00001) against protein databases (Nr, Swiss-Prot, KEGG and COG). ESTScan was used to predict 1708 coding sequences and translated them into peptide sequences for those unigenes with no BLAST hits. Besides, the CDS nucleic acid and protein sequences with ESTScan nucleic acid and protein sequences were obtained (Additional file 1: Figure S1).

### SNP identification and microsatellite

**Fig. 4:**
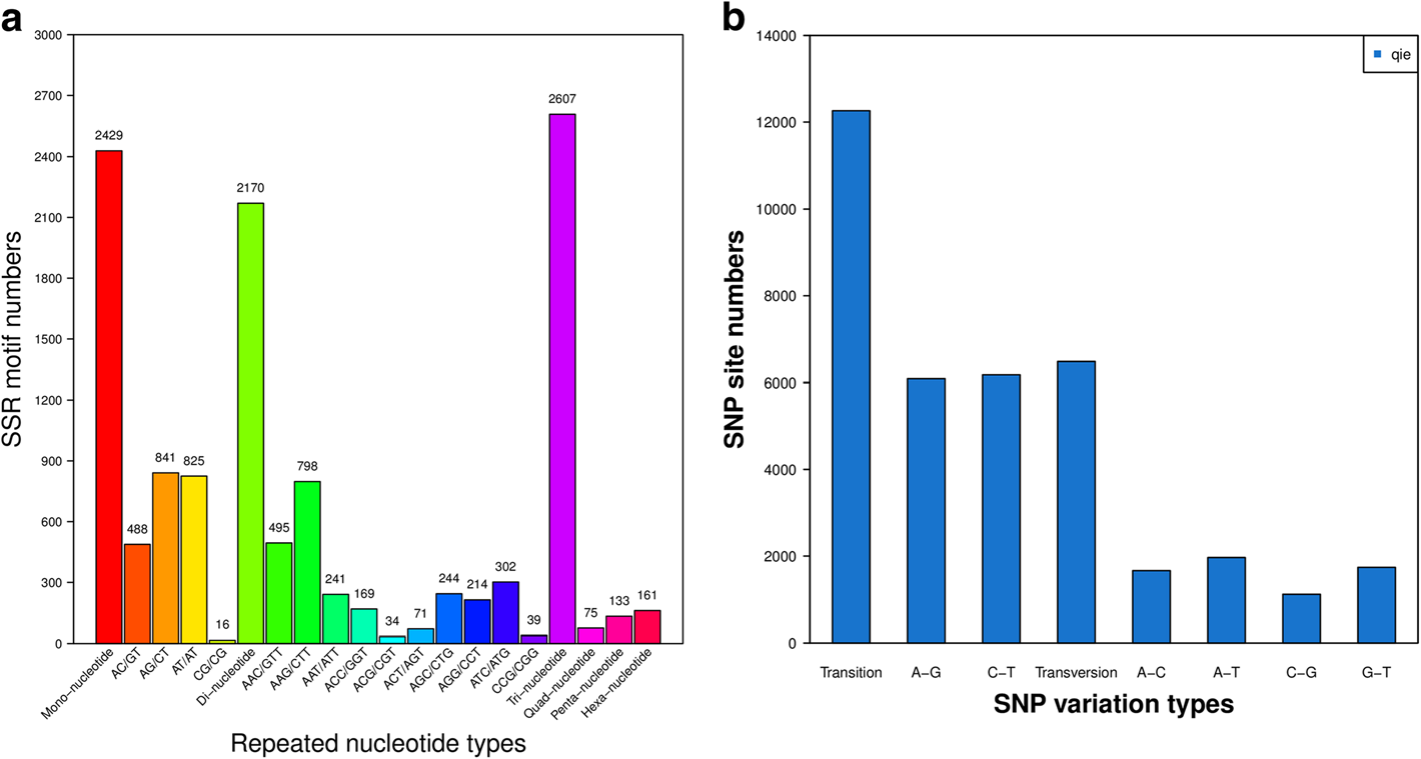
SNP identification and microsatellite. **a** Chart of SSR SSR length distribution. The X-axis is the repeat unit length and the specific repeat type, and the Y-axis is the SSR number. **b** Chart of SNP quantity statistics. The X-axis is the SNP type and the Y-axis is the SNP number

SSR detection of transcriptome was performed using Unigene as a reference sequence, further more, SSR software MicroSAtellite (MISA) was used to find all the SSR. The length of all SSR repeat units on Unigene was screened, and only keep the sequence which are not less than the 150 bp, with the design of primers. A total of 2607 SSRs were identified with up to tri-nucleotide (Fig. 4a).

We used SOAPsnp to detect the single nucleotide polymorphisms(SNP) of samples, it is a re-sequencing tool that can assemble the newly sequenced individuals on the reference sequence is blasted on the known sequencebe which are based on the original sequencing fragment. So that we can identify the single nucleotide polymorphisms passed by the consensus sequence and the reference sequence of the new sequencer on the reference sequence. In *Solanum aculeatissimum*, a total of 18,744 SNPs were identified, including transition 12,261 (A-G = 6086; C-*T* = 6175) and transversion 6483 (A-C = 1663; A-*T* = 1965; C-G = 1117; G-*T* = 1738) (Fig. 4b).

Overview of sRNA sequencing data sRNA analysis was performed using Unigene, which built assemblies from scratch to form a reference transcriptome. To identify the miRNAs involved in chilling tolerance (1 °C) in *S. aculeatissimum*, we constructed sRNA from 0 h, 2 h, 6 h, 12 h and 24 h samples using high-throughput sequencing (Berry Genomics Corporation, Beijing, China). We removed reads with no 3′ adapter and 5′ adapter, reads with no insertion, reads of less than 18 nt and reads containing polyA regions. This resulted in 12,890,133 (0 h), 13,699,236 (2 h), 9,796,428 (6 h), 9,979,347 (12 h) and 12,015,602 (24 h) clean reads corresponding to 5,192,942, 5,541,563, 4,132,312, 3,248,933 and 4,708,455 unique reads, respectively. These remaining reads were searched against the eggplant genome sequence, and 38.53%, 36.23%, 35.73%, 46.86% and 38.25% of the total sRNAs mapped to the genome, respectively. All sRNAs were compared to various RNAs using the GenBank database. GenBank comments were summarized, and the number and percentage of various sRNAs were listed (Table 2).

**Table 2:** Statistics of sRNA sequences from the five time periods

Types	oh		2h		6h		12h		24h	
	Total sRNAs	Unique sRNAs	Total sRNAs	Unique sRNAs	Total sRNAs	Unique sRNAs	Total sRNAs	Unique sRNAs	Total sRNAs	Unique sRNAs
Total	12890133 (100%)	5192942 (100%)	13699236 (100%)	5541563 (100%)	9796428 (100%)	4132312 (100%)	9979347 (100%)	3248933 (100%)	12015602 (100%)	4708455 (100%)
miRNA	132708 (1.03%)	14424 (0.28%)	136908 (1.00%)	15984 (0.29%)	121949 (1.24%)	15178 (0.37%)	128290 (1.29%)	14628 (0.45%)	154361 (1.28%)	18132 (0.39%)
rRNA	3098878 (24.04%)	236603 (4.55%)	3083593 (22.51%)	243551 (4.39%)	2142415 (21.87%)	206114 (4.99%)	3255035 (32.62%)	237080 (7.30%)	2881673 (23.98%)	237067 (5.03%)
rRNAetc	117385 (0.91%)	17333 (0.33%)	113499 (0.83%)	17849 (0.32%)	89695 (0.92%)	14946 (0.36%)	118167 (1.18%)	16783 (0.52%)	112693 (0.94%)	16859 (0.36%)
snRNA	3019 (0.02%)	1592 (0.03%)	2959 (0.02%)	1573 (0.03%)	1675 (0.025)	1001 (0.02%)	2655 (0.03%)	1159 (0.04%)	2313 (0.02%)	1178 (0.03%)
snoRNA	893749 (6.94%)	542973 (10.46%)	966614 (7.06%)	574651 (10.37%)	621241 (6.34%)	388387 (9.40%)	482064 (4.83%)	287530 (8.85%)	742203 (6.18%)	444084 (9.43%)
known_miRNA	586650 (4.55%)	2514 (0.05%)	662848 (4.84%)	2677 (0.05%)	643511 (6.57%)	2894 (0.07%)	880816 (8.83%)	3336 (0.10%)	779202 (6.49%)	3117 (0.07%)
tRNA	358701 (2.78%)	32239 (0.62%)	343134 (2.50%)	33004 (0.60%)	211015 (2.15%)	24598 (0.60%)	334856 (3.35%)	29742 (0.91%)	262192 (2.18%)	27983 (0.59%)
Un-annotated	7699043 (59.73%)	4345264 (83.68%)	8389681 (61.24%)	4652274 (83.95%)	5964927 (60.89%)	3479194 (84.19%)	4777464 (47.87%)	2658675 (81.83%)	7080965 (58.93%)	3960035 (84.10%)
Mapping to genome	4967104 (38.53%)	594810 (11.45%)	4962707 (36.23%)	595219 (10.74%)	3500382 (35.73%)	439554 (10.64%)	4676439 (46.86%)	406764 (12.52%)	4595584 (38.25%)	499205 (10.6%)

**Fig. 5:**
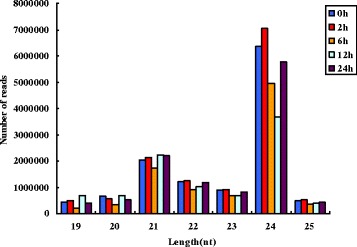
Length distribution of sRNAs in five time periods. Different colors represent different time periods, from left to right followed by 0 h, 2 h, 6 h, 12 h, 24 h

The length distribution of the sRNAs from the five libraries show that the proportion of 19–25 nt sequences was high, comprising over 85% of the sequences. sRNAs of 21 and 24 nt in length were the two main classes among the sequences (Fig. 5). Among unique sRNA sequences, 24 nt was the most abundant length, which was in agreement with previous reports on cucumber [23], grapevine [24] and rice [25].

### Identification of conserved miRNAs

**Fig. 6:**
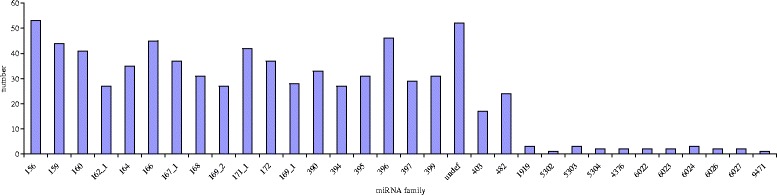
Numbers of conserved miRNAs families in different plant species

To identify miRNAs involved in chilling tolerance in *S. aculeatissimum*, we used high-throughput sequencing to filter out sRNA sequences after 0 h, 2 h, 6 h, 12 h, and 24 h. Furthermore, we read and classified the RNA and annotated the miRNAs, which were identified by comparing sRNAs to the miRBase database by BLAST or BOWTIE, and these known miRNAs were used for subsequent analysis. This resulted in 306 conserved miRNAs belonging to 31 miRNA families. Interestingly, the miRNA families exist differently in other species (Fig. 6). The largest family was the 156 miRNA family that exist in 53 species, while the 5302 and 9471 miRNA families were the smallest, only exist in one species. A family analysis of detected miRNAs was conducted to explore the presence of miRNA families in the other species and the conservation of these miRNAs. The selected species were sequenced for comparison with plants with known genomic sequences.

### Identification of novel miRNAs

In this study, MIREAP was used to predict new un-annotated miRNAs and compare these to sRNAs in the antisense, intron and intergenic regions of the genome exome and to map the secondary structure of these new miRNAs. miRNA precursors have a characteristic stem-loop hairpin secondary structure, which is one of the most important features for separating miRNA from endogenous sRNA. The method is part of screening for cold-resistant miRNAs.

At the same time, we compared specific lengths of sRNA to the eggplant reference genome sequence for prediction and sequence analysis of new miRNAs. In total, 60, 70, 72, 73 and 78 new miRNAs were predicted at 0, 2, 6, 12 and 24 h, respectively. In addition, we predicted the secondary structure of these new miRNAs (Additional file 2: Table S1.1, Additional file 3: Figure S2). At the same time, miRNAs and target genes occurred in at least two sets of differential expression simultaneously, finally 11 novel miRNAs and 108 target genes were identified (Additional file 2: Table S1.2).

The base composition of the nucleotide sequence may affect its physical or biochemical properties. Analyzing the first base of miRNAs is important for assessing the quality of sequencing and the biological function of miRNAs. The distribution of the first base has a strong bias for different lengths of miRNAs. The proportion of base U is the largest, especially in 20–24 nt miRNAs, followed by base A. Bases G and C are relatively infrequent in the first position of miRNA (Additional file 4: Figure S3).

### Cold tolerance candidate miRNAs and target genes

As previously mentioned, miRNAs promote the degradation of target mRNA or translation inhibition by binding to the 3′ untranslated region. This is the mode of action of plant miRNAs, and it depends on the degree of complementarity of the miRNA to its target site [26]. In general, miRNAs are complementary or nearly completely complementary to the target mRNA and will cleave the target mRNA when they are not fully complementary, thereby inhibiting target mRNA translation [27, 28]. Unlike mammalian miRNAs, plant miRNA-mediated target mRNA cleavage is the dominant mode of action. In general, plant miRNAs act in a manner similar to small interfering RNAs (siRNAs). They are precisely cleaved at base 10 or 11 of the miRNA after binding to a specific site in the target mRNA coding region in a fully complementary or nearly fully complementary manner, leading to degradation of target mRNA [29–32]. Mutations associated with the miRNA pathway lead to a significant decrease in miRNA levels, affecting miRNA cleavage and a significant increase in target mRNA levels [33, 34].

In a broad sense, sRNA and mRNA association analysis can be understood as miRNAs regulating multiple mRNAs and being regulated at a number of levels, including at post-transcriptional levels by different biological and metabolic processes and a variety of biological activities or related diseases. In this study, five time points were analyzed, which allowed investigation of changes over 0–2 h, 0–6 h, 0–12 h, 0–24 h, 2–6 h, 6–12 h and 12–24 h (Additional file 5: Figure S4). We identified 56 down-regulated miRNAs and 28 up-regulated miRNAs in *S. aculeatissimum*, corresponding to 220 up-regulated mRNAs and 94 down-regulated mRNAs, respectively (Additional file 6: Table S2). At the same time, we screened nine significant differentially expressed miRNAs and their mRNAs by association analysis (Additional file 7: Table S3). Among these, we focused on the analysis of nine miRNAs and 12 target genes (Table 3). The expression differences of miR168a, miR2652a, miR812v, miR4414a-5p and miR5813 and their target genes occurred in two groups, and the expression differences of miR167c-3p, miR9478-3p, miR4221 and miR8577 and their target genes occurred in four groups(0–2 h, 0–6 h, 0–12 h and 0–24 h).

**Table 3:** Significant targets of miRNAs match different time periods

miRNA	Target geneID	Target gene description
miR168a	CL2784.Contig3_qie	PREDICTED: probable bifunctional methylthioribulose-1-phosphate dehydratase/enolase-phosphatase E1 1-like [*Solanum lycopersicum*]
miR2652a	CL4812.Contig2_qie	PREDICTED: uncharacterized protein LOC101252455 [Solanum lycopersicum]
miR812v	CL3453.Contig3_qie	PREDICTED: xylogalacturonan beta-1,3-xylosyltransferase-like [Solanum lycopersicum]
miR4414a-5p	CL10904.Contig2_qie	squamosa promoter binding-like protein [Solanum lycopersicum]
	Unigene13436_qie	protein phosphatase 2C [Solanum lycopersicum] > gi|46,277,128|gb|AAS86762.1| protein phosphatase 2C [Solanum lycopersicum]
miR5813	CL2972.Contig2_qie	PREDICTED: uncharacterized protein LOC101255162 [Solanum lycopersicum]
	Unigene14085_qie	Ser/Thr protein kinase [*Solanum chacoense*]
miR167c-3p	CL6300.Contig1_qie	PREDICTED: ATP sulfurylase 1, chloroplastic-like [Solanum lycopersicum]
miR9478-3p	CL5359.Contig1_qie	PREDICTED: mitogen-activated protein kinase kinase kinase A-like [Solanum lycopersicum]
	CL8722.Contig1_qie	PREDICTED: uncharacterized protein LOC101255771 [Solanum lycopersicum]
miR4221	CL8275.Contig1_qie	Putative retrotransposon protein, identical [*Solanum demissum*]
miR8577	CL9325.Contig1_qie	PREDICTED: putative glycerophosphoryl diester phosphodiesterase YhdW-like [Solanum lycopersicum]

### qRT-PCR verification

To examine miRNA expression levels and verify the sequencing results, nine miRNAs were selected for further analysis. First, we found the sequence of mature miRNAs from the miRNA database miRbase, and then we used these sequences to design miRNA-specific reverse transcription stem-loop primers, different upstream primers and universal downstream primers. The stem loop qRT-PCR method was used with the classic stem-loop primer 5′-GTTGGCTCTGGTGCAGGGTCCGAGGTATTCGCACCAGAGCCAAC-3′ (Additional file 8: Table S4.1) [35]. Primers for target genes were designed using Primer 5.0 (Additional file 8: Table S4.2).

**Fig. 7:**
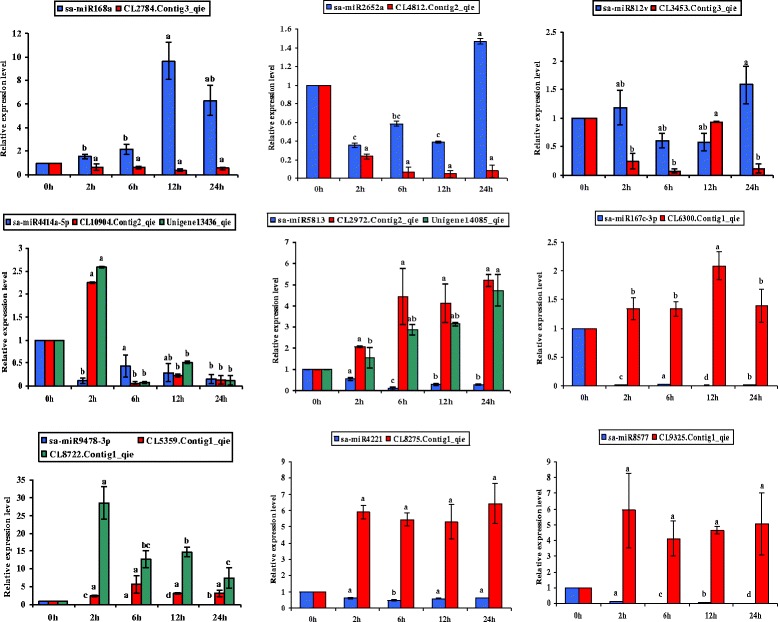
qRT-PCR validation of miRNAs and target genes in the wild eggplant at control (0 h) or low temperature(2 h, 6 h, 12 h and 24 h). Small nuclear RNAU6 is used as an internal reference. Action is used as an internal reference for target genes. The data of target genes are the average of three qRT-PCR replicates for each sample from three biological repeats. Error bars indicate one standard deviation of three different qRT-PCR replicates

Among these conserved miRNAs, the expression of sa-miR168a, sa-miR2652a, sa-miR812v and sa-miR4414a-5p generally increased under low temperature, which was a significant difference from the control. The expression of sa-miR5813, sa-miR167c-3p, sa-miR9478-3p, miR4221 and miR8577 generally decreased under low temperature. qRT-PCR was also performed on 12 related target genes that interactive expression with 9 miRNAs (Fig. 7). We could see that miRNAs show negative regulation of target genes from qRT-PCR.

### Functional analysis of miRNA targets in eggplant

To evaluate the effects of low-temperature stress on miRNA expression, we compared the expression between control and low-temperature stress-treated groups over five time periods. To improve the accuracy, down-regulated miRNA that corresponded to up-regulated target genes and up-regulated miRNA that corresponded to down-regulated target genes were selected. After using differences in the expression of miRNA target genes to select from the 80 most apparent targets, nine miRNAs that significantly changed under low-temperature stress were chosen for further analysis.

Expression analysis of miRNA target genes based on differential expression of transcriptome mRNA, allowed us to identify differentially expressed genes and enrichment analysis of the target gene function. First, mapped all differentially expressed genes to various terms in the Gene Ontology database (http://www.geneontology.org/) and KEGG Pathway database (http://www.genome.jp/kegg/pathway.html), calculated the number of genes for each term, and then applied the hypergeometric test to find out whether GO and KEGG are significantly enriched in differentially expressed genes by compared to the entire genome background. Because miRNA is a negative regulator, both up-regulation of miRNAs and down-regulation of mRNAs, as well as down-regulation of miRNAs and up-regulation of mRNAs, were analyzed. GO (*p*-value ≤0.05) and KEGG (p-value ≤0.05) enrichment analysis were performed to identify expression and regulatory pathways involved in differentially expressed genes. GO is an internationally standardized gene functional classification system that comprehensively describes the properties of genes and gene products in organisms. GO has a total of three ontology, respectively, molecular function (MF), cellular position (CC), biological process (BP). MF describes the activity in molecular biology, such as catalytic activity or binding activity. The CC refers to the group of organelles or gene products in which the gene product is located. BP is a process that consists of multiple steps of molecular function, broadly like cell growth and maintenance, signaling. We then analyzed gene function (Additional file 9: Table S5).

As a result, we identified 56 down-regulated miRNAs and 28 up-regulated miRNAs in *S. aculeatissimum*, corresponding to 220 up-regulated mRNAs and 94 down-regulated mRNAs, respectively. At the same time, we screened the nine miRNAs with the most significant differences in chilling tolerance from *S. aculeatissimum* by qRT-PCR to explore genes that regulate cold tolerance mechanisms.

## Discussion

### Conserved and novel miRNAs in *S. aculeatissimum* at the low temperatures

miRNAs are a class of non-coding, that are important regulators involved in plant growth, development and stress responses. miRNAs have received increasing amounts of attention in tomato [36–38], tea plants(*Camellia Sinensis*) [39], grapevines [40], cassava [41] and *Populus tomentosa* [42]. In recent years, a new approach for discovering miRNAs using high-throughput sequencing technology has been widely used to identify conserved and novel miRNAs in plants This has enlarged the scope of miRNA research and made miRNAs a hotspot of epigenetic research.

The eggplant is an important vegetable crop that is grown worldwide. Chilling is a common abiotic stress that affects eggplant cultivation, especially during the winter in greenhouses. Since miRNAs are thought to be important for the abiotic stress response and a high-through sequencing approaches are available, research on chilling-responsive miRNAs in eggplants will help to reveal the response mechanisms of these miRNAs and to further elucidate the mechanisms of miRNA regulation in general. More importantly, this study provides a theoretical foundation for further research on miRNA regulation of the response to chilling stress.

Of the sRNAs identified in our study, 24-nt sRNAs were the most abundant (Fig 5), comprising 306 conserved miRNAs belonging to 31 miRNA families (Fig 6). After screening and removal of redundant reads, 56 down-regulated miRNAs and 28 up-regulated miRNAs were identified in *S. aculeatissimum*, corresponding to 220 up-regulated mRNAs and 94 down-regulated mRNAs, respectively.

### Cold-responsive miRNAs and target genes in *S. aculeatissimum* at the low temperatures

In this study, miRNAs that were significantly up- or down-regulated when the eggplants were exposed to low temperatures were considered to be cold-responsive. In summary, eighty-forth miRNAs responded to low temperatures, fifty-six of these were down-regulated, and twenty-eight were up-regulated.

Cold-related miRNAs have been validated using other plant models [43–45]. In *Chorispora bungeana,* the expression of miR168 increased under chilling-stress, and this study obtained similar results [43]. In other abiotic stresses, such as drought stress, miR812 shows increased expression in *indica* rice var. Nagina22 [46]. Yongbin Ou et al. found that stu-miR4414 was down-regulated in the frozen potato tubers [47], which is similar to our results in *S. aculeatissimum*. Interestingly, miR5813 was also found to be involved in cold stress in Sugarcane [48]. In tomato, miR5813 was also verified to be involved in environmental stress [49]. In the analysis of Arabidopsis response to abiotic stress, M Barciszewskapacak pointed out that miR167a-3p response to heat stress, indicating that miR167 has a certain correlation with temperature stress [50]. At the same time, The expression of sa-miR168a, sa-miR2652a, sa-miR812v, sa-miR5813, sa-miR167c-3p, sa-miR9478-3p, sa-miR4221 and sa-miR8577 are similar to the sequencing results. These indicate that high-throughput sequencing is reliable and that these miRNAs have a regulatory mechanism in response to eggplant stress in response to cold stress.

We performed qRT-PCR on at least one target gene for each miRNA and showed that mRNA and miRNA had the opposite expression, indicating that miRNAs have a negative regulatory mechanism on the target gene.

### Analysis of miRNAs and target genes at the low temperatures

Among the candidate cold-adapted miRNAs and their target genes, we screened the more important 9 miRNAs and 12 target genes. Among them, miR168a, miR2652a, miR812v and miR4414a-5p were up-regulated under low temperature stress, while miR5813, miR167c-3p, miR9478-3p, miR4221 and miR8577 were down-regulated. We divided the five time periods of cryogenic treatment into seven groups (0–2 h, 0–6 h, 0–12 h, 0–24 h, 2–6 h, 6–12 h and 12–24 h) for comparison. The results showed that miR168a was differentially expressed at 0–6 h and 0–12 h, besides its target gene was significant difference at 0–2 h and 0–6 h, miR2652a was similar with miR168a, its target gene was differentially expressed at 0–2 h and 0–12 h. Among them, the expression of miR812v increased significantly just under low temperature stress. Interestingly, miR4414a-5p and miR5813 showed similar expression changes under low temperature stress, miR4414a-5p was up-related while miR5813 was down-related. We obtained that miR167c-3p, miR9478-3p, miR4221 and miR8577 showed significant changes under low temperature stress, with obvious down-regulation in all four time periods (0–2 h, 0–6 h, 0–12 h, 0–24 h).

At the same time, miRNAs and their target genes showed a negative regulatory response, which was consistent with the result of qRT-PCR. As can be seen from Fig. 7, the expression of sa-miR168a was inhibited under low temperature stress, the expression of sa-miR168a decreased with the negative regulation of the CL2784.Contig3_qie expression increased, and the expression was highest at 12 h. We analyzed sa-miR5813 and its target genes CL2971.Contig2_qie and Unigene14085_qie, the expression of sa-miR5813 decreased under stress, while its target genes CL2971.Contig2_qie and Unigene14085_qie showed a similar increase. In addition, the expression of sa-miR9478-3p was similar with sa-miR5813. We also analyzed the similarities of sa-miR167c-3p, sa-miR4221 and sa-miR8577, three miRNAs showed down-regulated significantly under cold stress and correlation at five time points, meanwhile, the target genes CL6300.Contig1_qie, CL8275.Contig1_qie and CL9325.Contig1_qie were up-regulated respectively.

## Conclusions

In summary, hundreds of chilling-responsive miRNAs have been identified in different plants using high-throughput sequencing, but fewer have been identified in eggplants. This information fills gaps in the knowledge of eggplant chilling-responsive miRNAs and increases the knowledge of miRNA responses to stress. Additionally, many target genes of miRNAs were characterized. The results showed that target genes were involved in various functions, including the expression of anti-stress proteins and antioxidant enzymes, among others. These findings lay a foundation for exploring the role of miRNA regulation of the eggplant response to chilling stress.

## Additional files

Additional file 1: Figure S1. Protein-Coding Region Prediction. (DOCX 237 kb)

Additional file 2: Table S1. New miRNA prediction and sequence analysis. Significant novel miRNAs match different time periods. (XLSX 71 kb)

Additional file 3: Figure S2. The secondary structure of novel miRNAs m0001. (DOCX 38 kb)

Additional file 4: Figure S3. The distribution of the first base has a strong bias for different lengths of miRNAs. (DOCX 196 kb)

Additional file 5: Figure S4. Figures of miRNA and gene differential expression. A: miRNA differential expression; B: Gene differential expression. (DOCX 559 kb)

Additional file 6: Table S2. Targets of conserved miRNAs from eggplant. Chi-square test and fisher-exact test results were both <0.05 and | log2 (CT/NT) | ≥ 1. (XLSX 54 kb)

Additional file 7: Table S3. Analysis of nine miRNAs and their genes differential expression. Chi-square test and fisher-exact test results were both <0.05 and | log2 (CT/NT) | ≥ 1. (XLSX 15 kb)

Additional file 8: Table S4. Sequences of primers used for stem-loop qRT-PCR analysis. (XLSX 11 kb)

Additional file 9: Table S5. Functional analysis of miRNA and target genes in eggplant. GO enrichment analysis of the functions of target genes cleaved by conserved miRNAs. KEGG pathway analysis of target genes of conserved miRNAs in different time periods. (XLSX 36 kb)

## Abbreviations

miRNAs: MicroRNAs
qRT-PCR: Quantitative Real-time PCR
*S. aculeatissimum*: *Solanum aculeatissimum*
sRNA: Small RNA
