# Supplementary material for: Cold-responsive miRNAs and their target genes in the wild eggplant species Solanum aculeatissimum
Source: BMC Genomics. 2017 Dec 29;18:1000. doi: 10.1186/s12864-017-4341-y (PMC5747154; doi:10.1186/s12864-017-4341-y)
Supplement: Supplementary file 1 — Protein-Coding Region Prediction. (DOCX 237 kb) [file 12864_2017_4341_MOESM1_ESM.docx]

**Figure S1 Protein-Coding Region Prediction**

**A. qie-Unigene.blast.cds.fa.length**

**
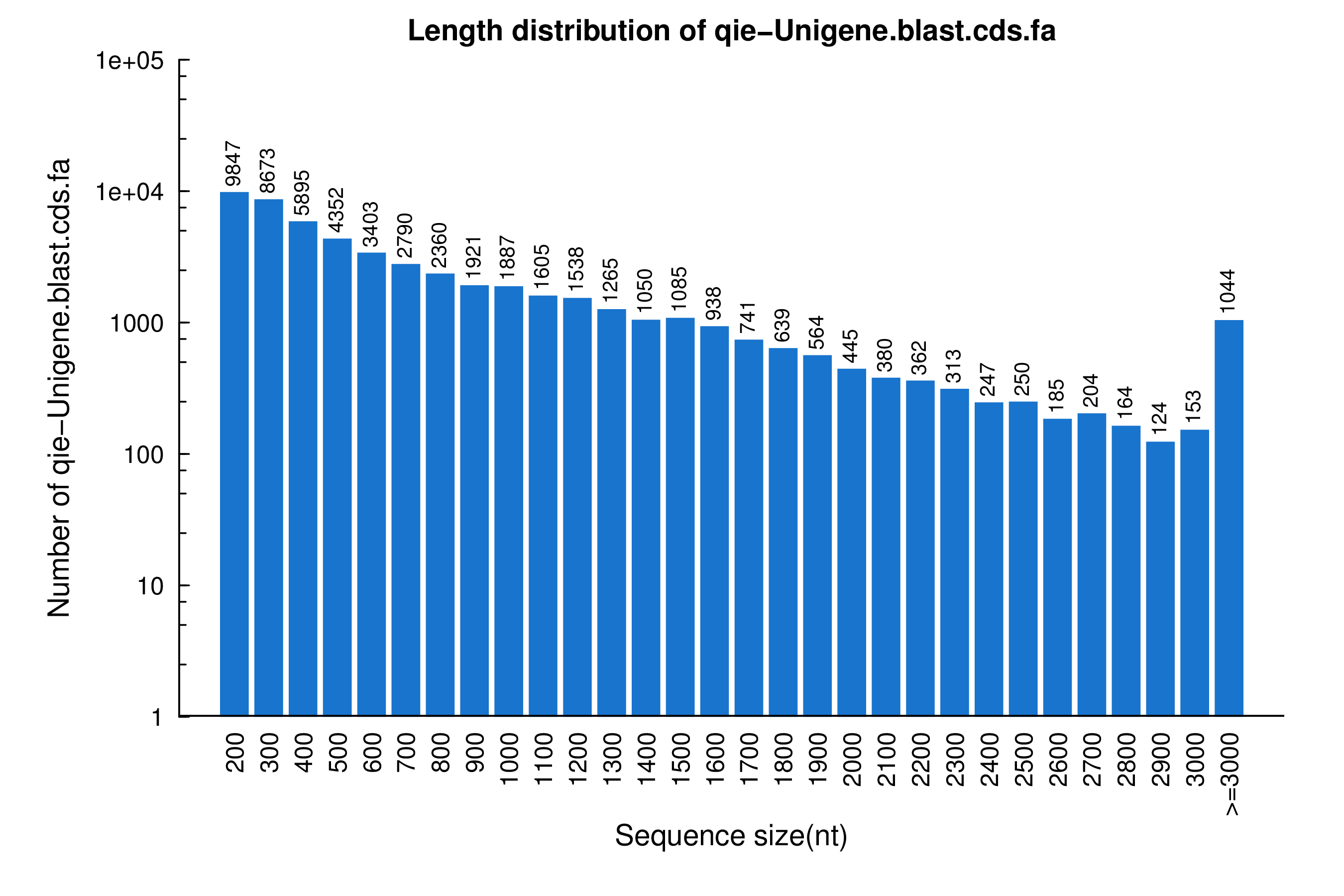
**

**B. qie-Unigene.ESTscan.cds.fa.length**

**
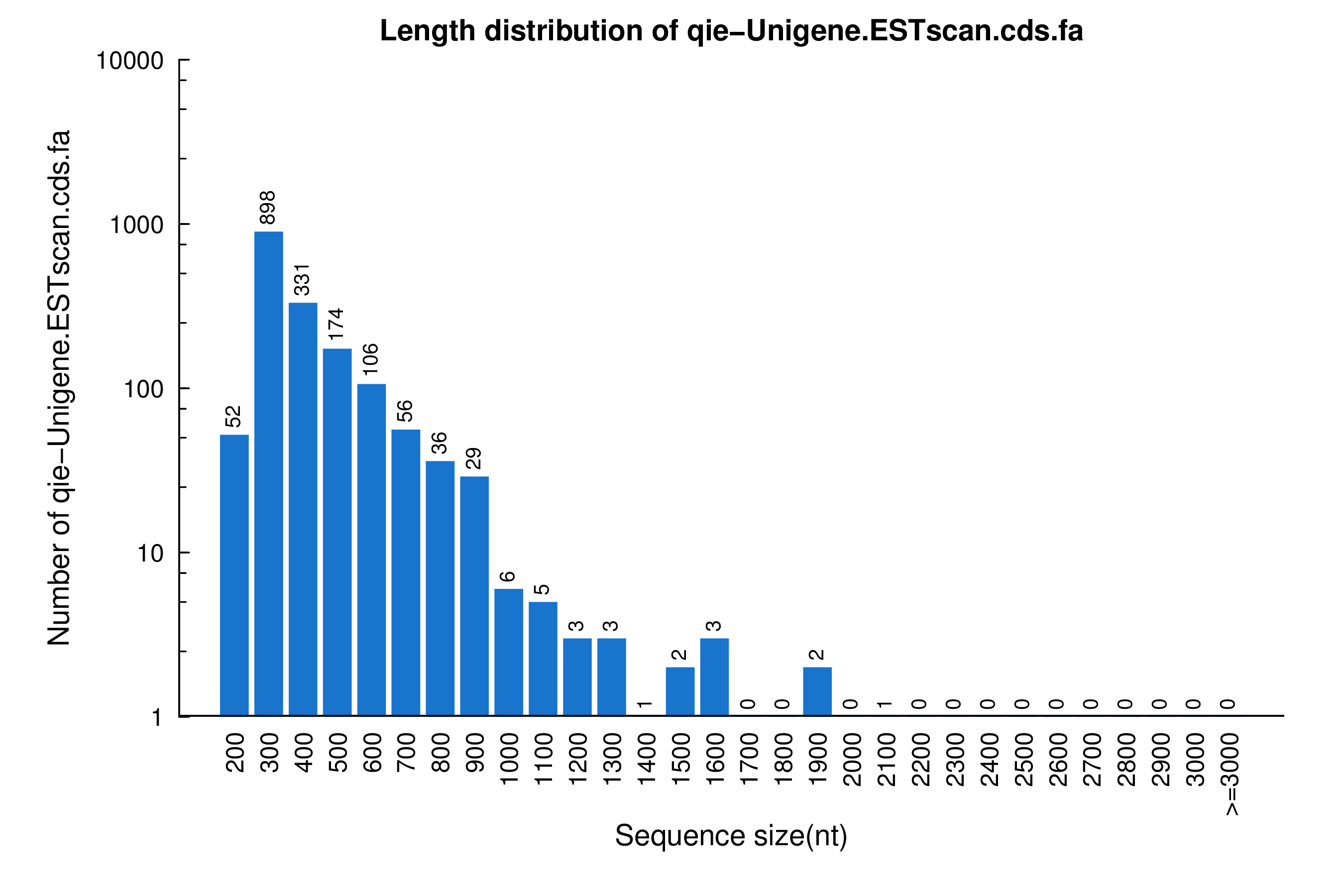
**

**Note: The abscissa is the length of the nucleic acid, and the ordinate is the number of nucleic acids corresponding to the length.**

**C. qie-Unigene.blast.protein.fa.length**

**
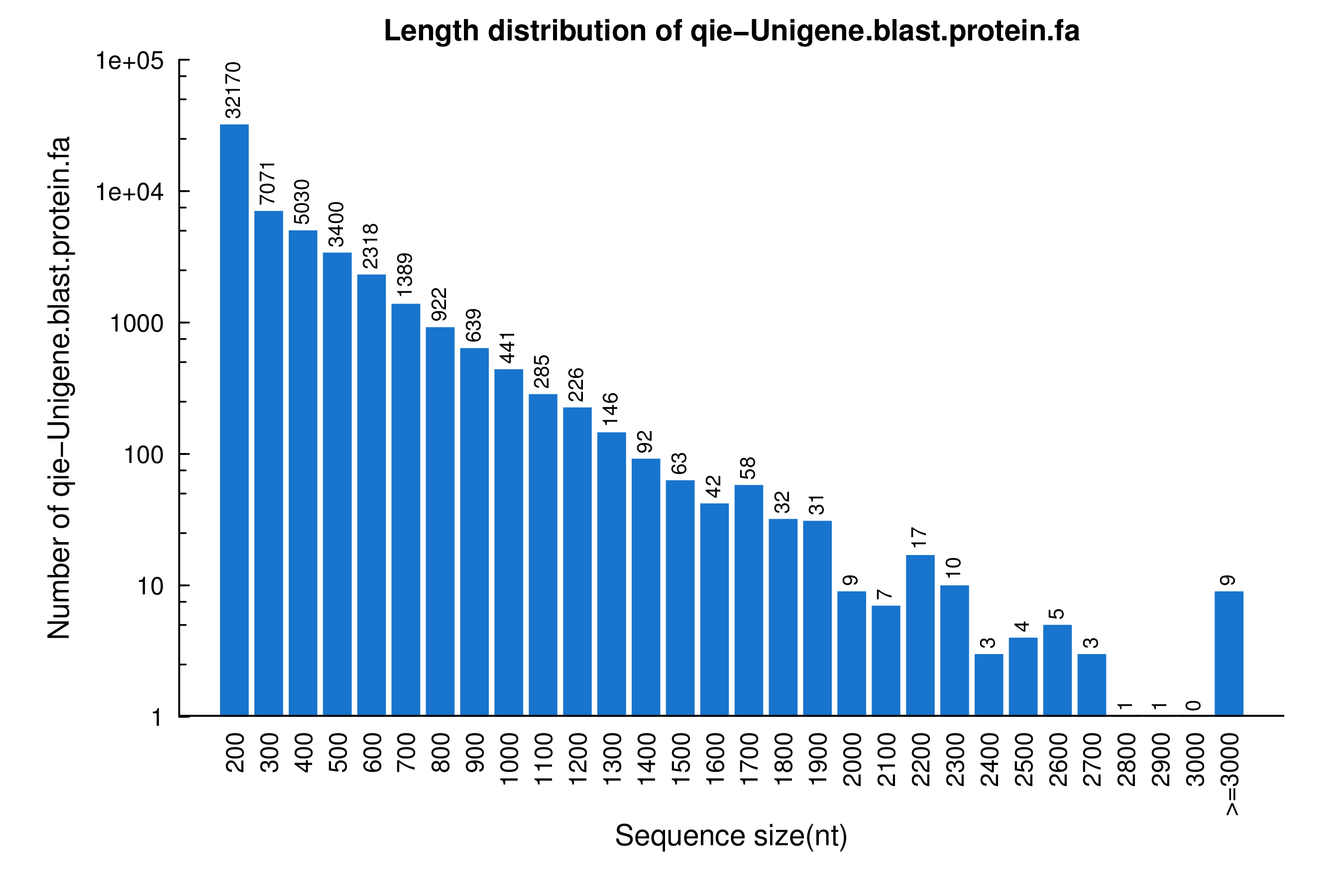
**

**D. qie-Unigene.ESTscan.protein.fa.length**

**
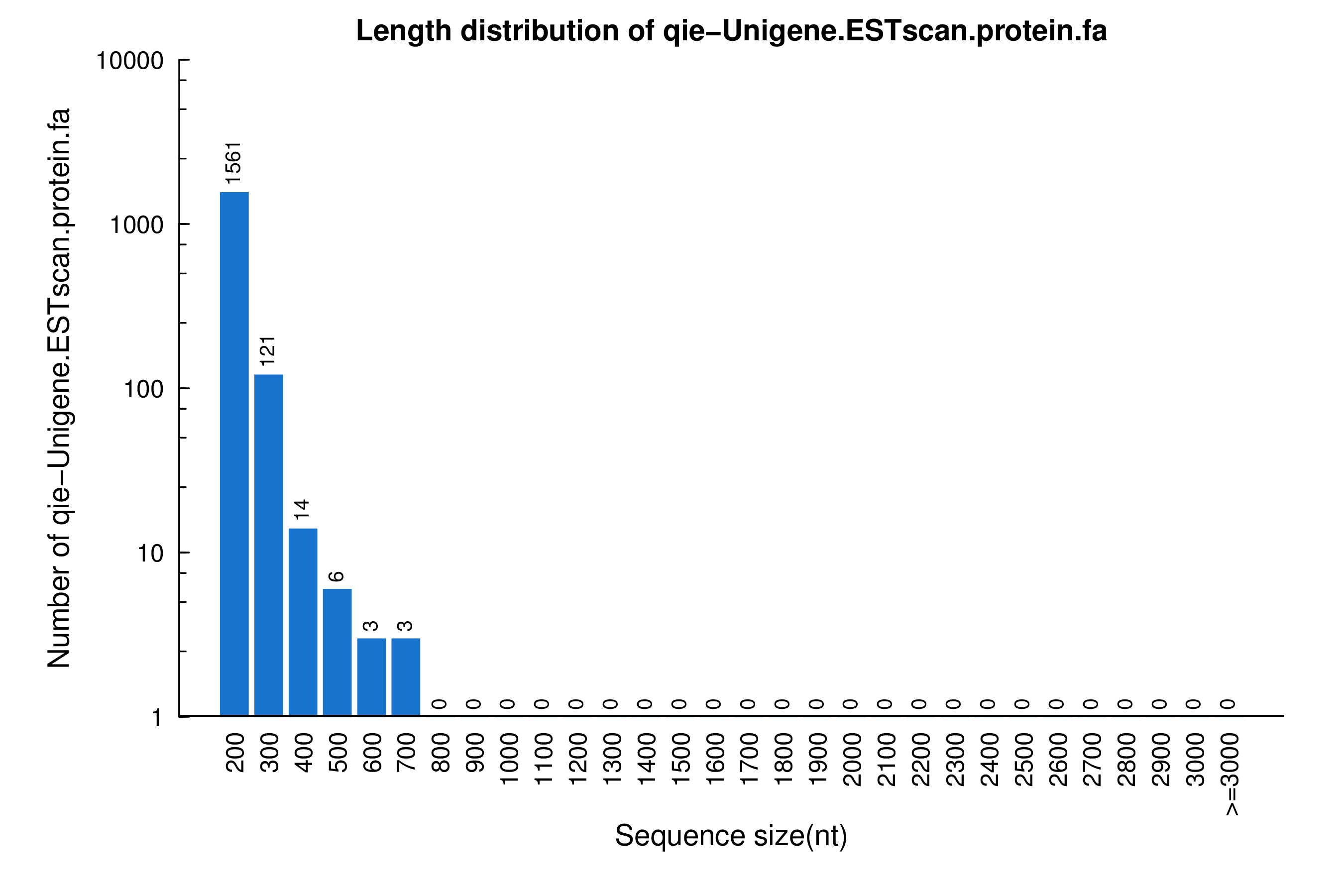
**

**Note: The abscissa is the length of the protein, and the ordinate is the number of proteins corresponding to the length.**
