# Supplementary figures and images for: Cold-responsive miRNAs and their target genes in the wild eggplant species Solanum aculeatissimum
Source: BMC Genomics. 2017 Dec 29;18:1000. doi: 10.1186/s12864-017-4341-y (PMC5747154; doi:10.1186/s12864-017-4341-y)

**Figure S2 m0001 secondary structure in five time periods**


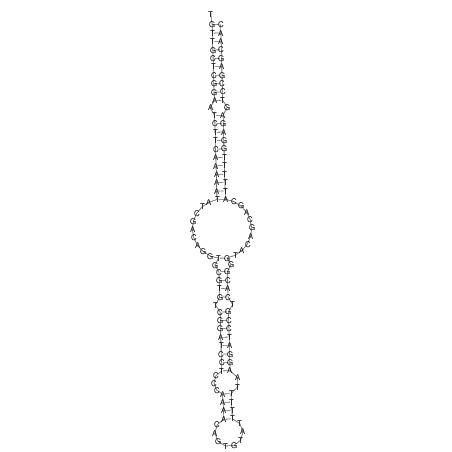


0h-m0001


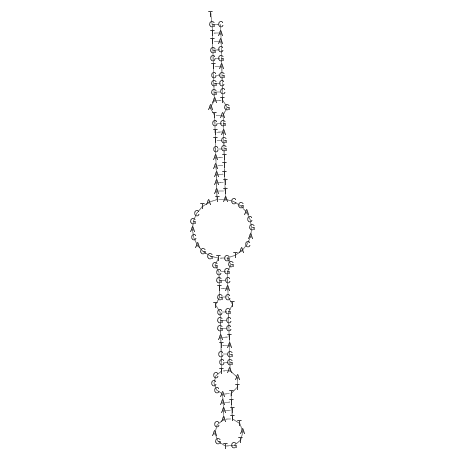


2h-m0001


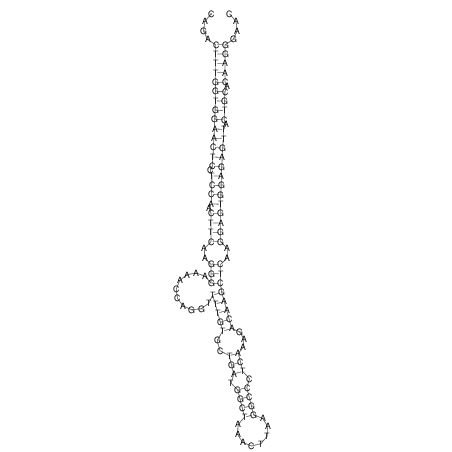


6h-m0001


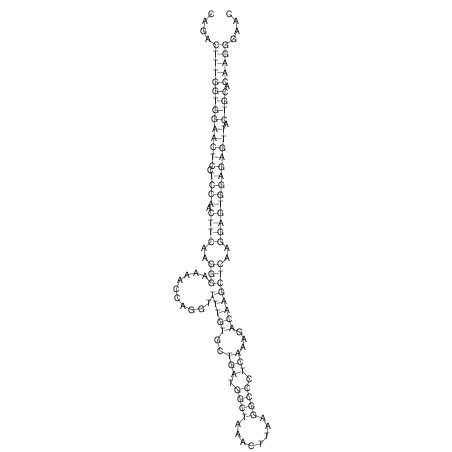


12h-m0001


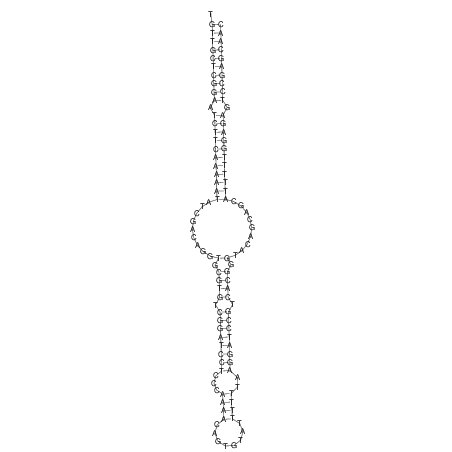


24h-m0001

Supplement: Supplementary file 3 — The secondary structure of novel miRNAs m0001. (DOCX 38 kb) [file 12864_2017_4341_MOESM3_ESM.docx]

**Figure S3 The base bias in five time periods**

**
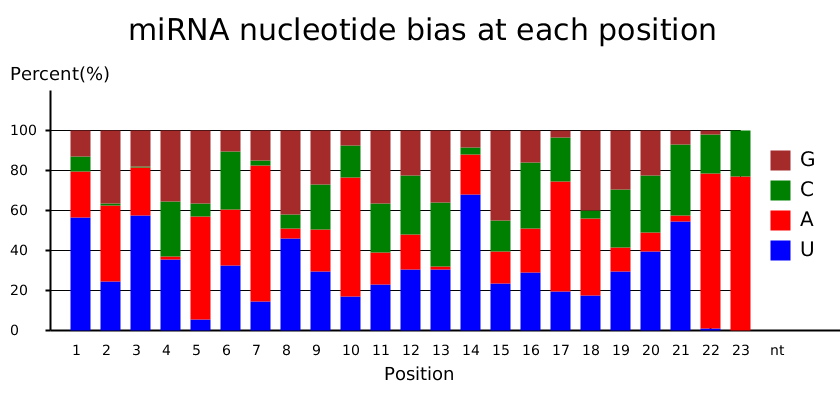
**

**
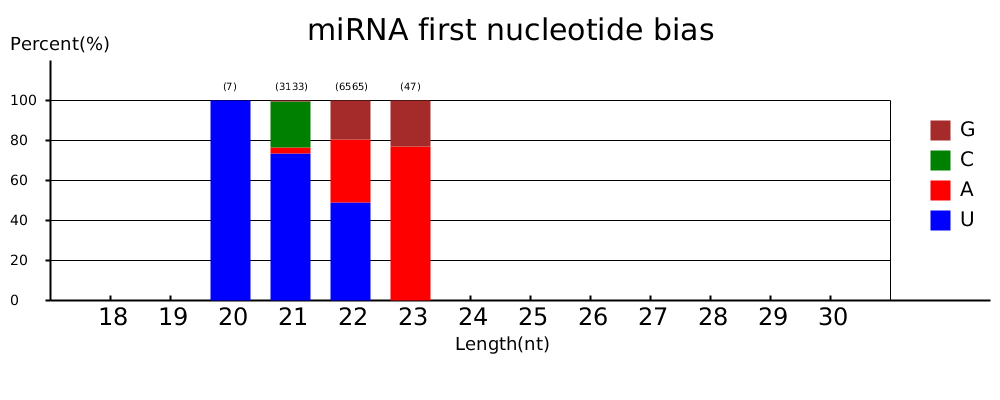
**

**0h**

**
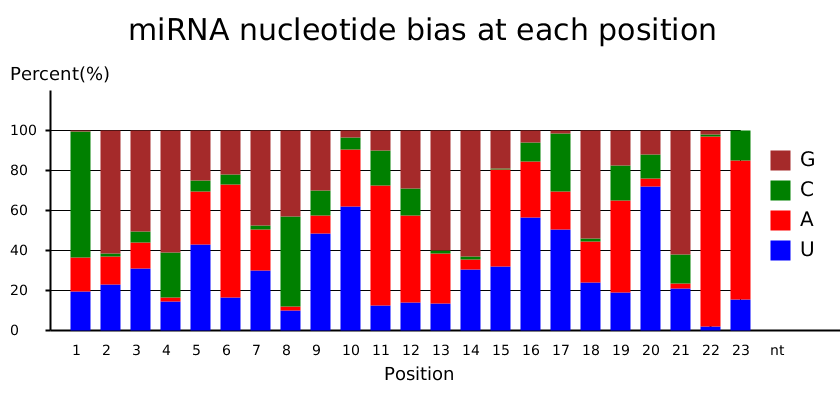
**

**
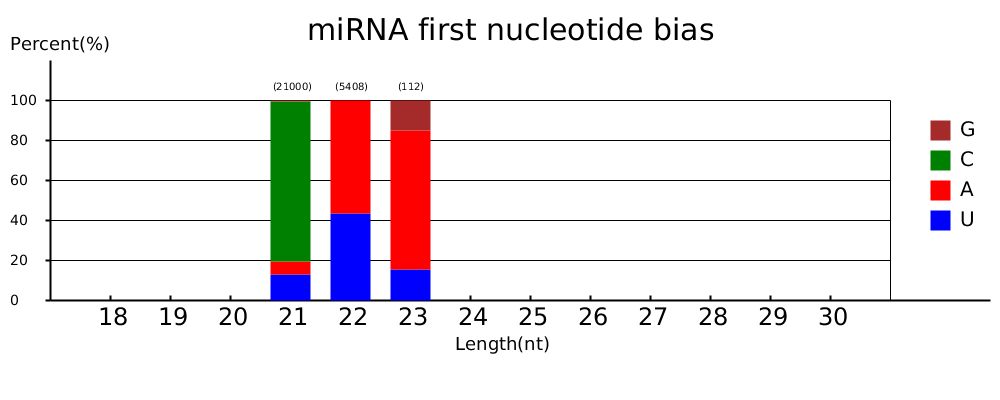
**

**2h**

**
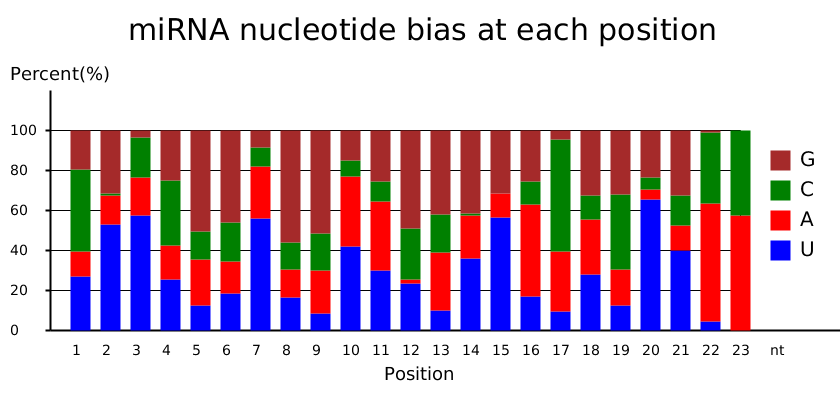
**

**
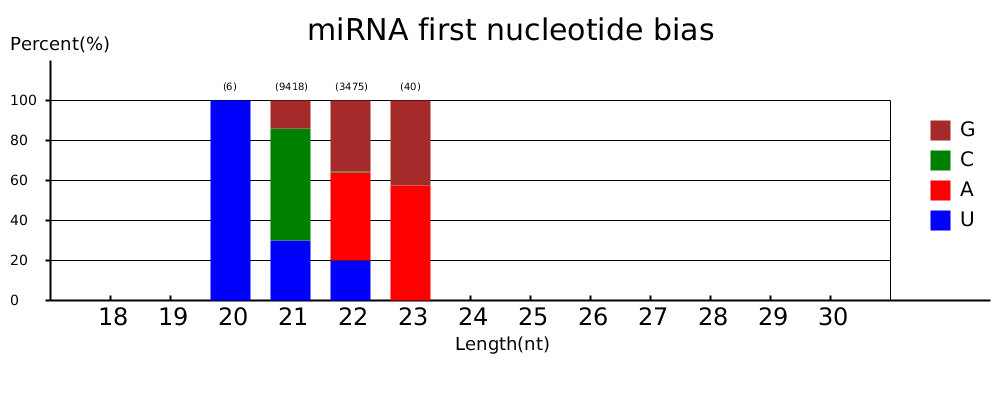
**

**6h**

**
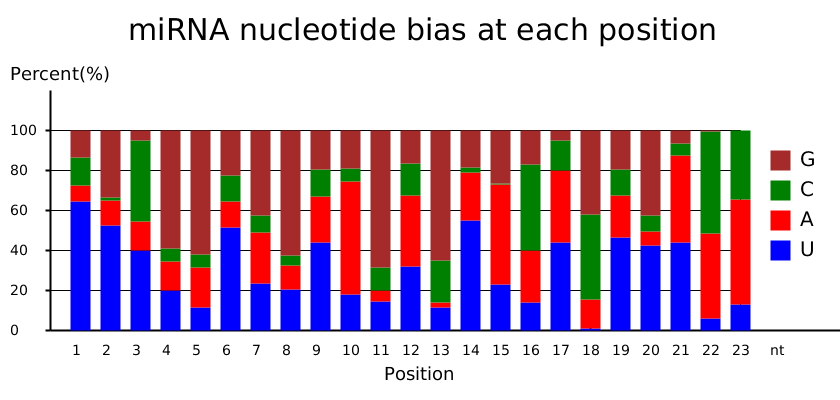
**

**
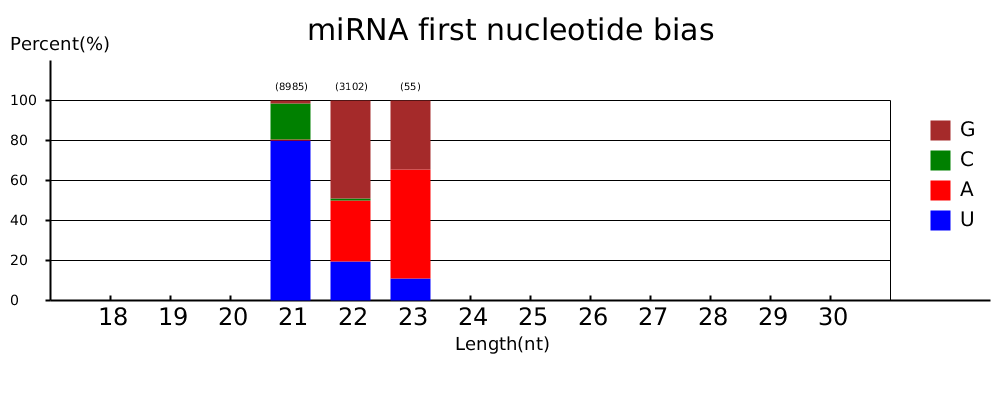
**

**12h**

**
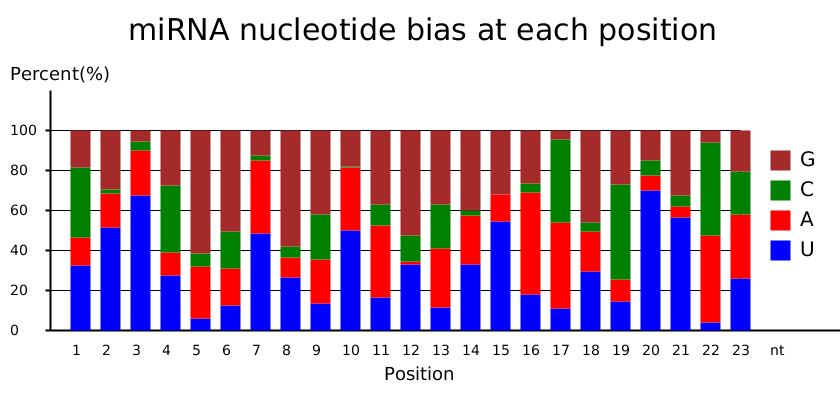
**

**
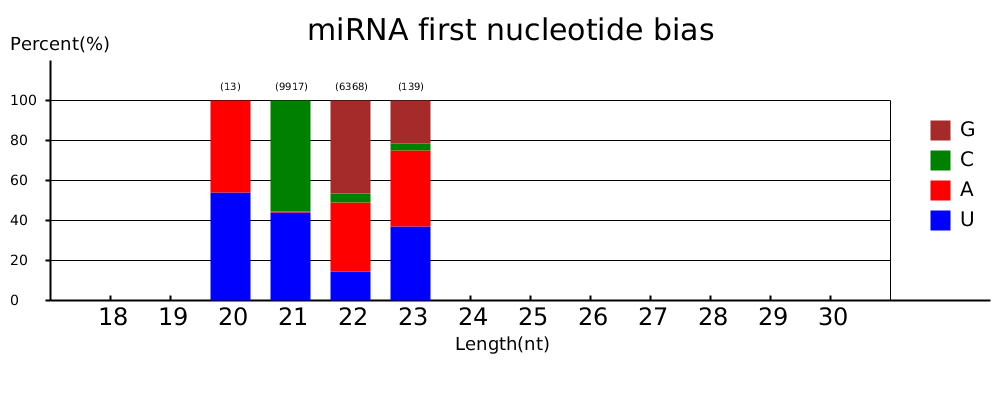
**

Supplement: Supplementary file 4 — The distribution of the first base has a strong bias for different lengths of miRNAs. (DOCX 196 kb) [file 12864_2017_4341_MOESM4_ESM.docx]
