# Supplementary material for: Cold-responsive miRNAs and their target genes in the wild eggplant species Solanum aculeatissimum
Source: BMC Genomics. 2017 Dec 29;18:1000. doi: 10.1186/s12864-017-4341-y (PMC5747154; doi:10.1186/s12864-017-4341-y)
Supplement: Supplementary file 5 — Figures of miRNA and gene differential expression. A: miRNA differential expression; B: Gene differential expression. (DOCX 559 kb) [file 12864_2017_4341_MOESM5_ESM.docx]

**Figure S4 Analysis of miRNA and gene differential expression**

**A: miRNA differential expression**

**
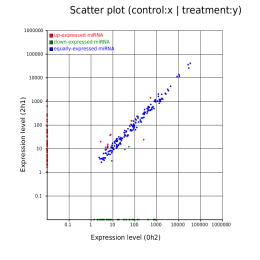

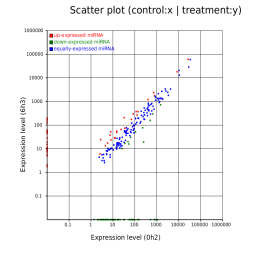

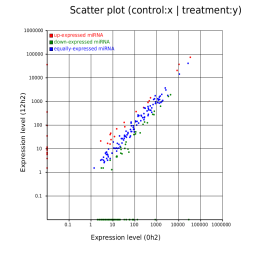

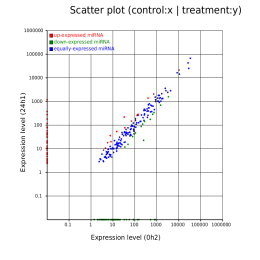
**

**0h-2h 0h-6h 0h-12h 0h-24h**

**
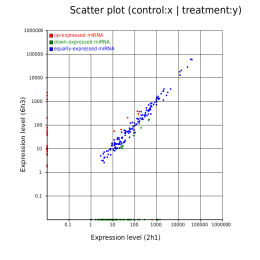

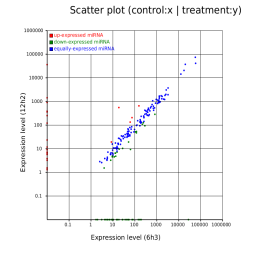

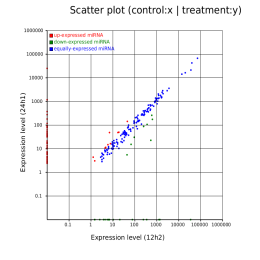
**

**2h-6h 6h-12h 12h-24h**

**B: Gene differential expression**

**0h-2h**

**
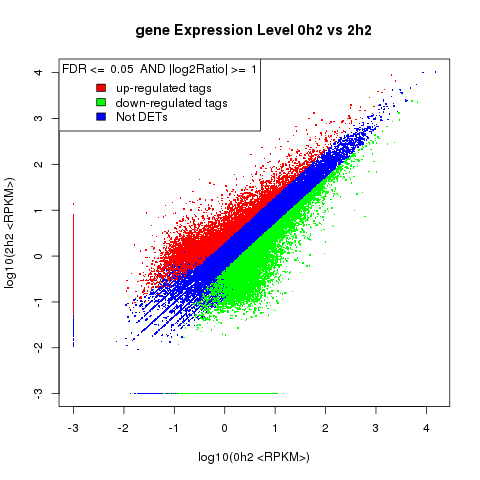

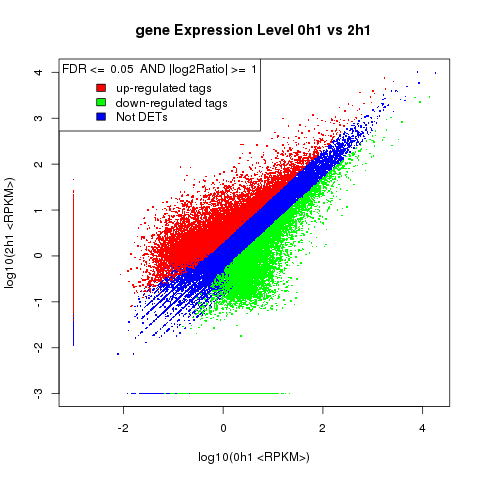

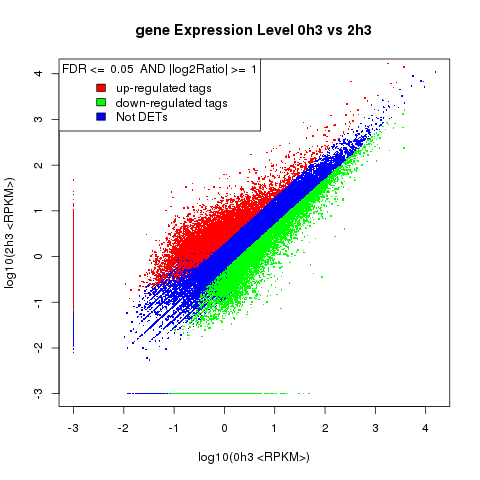
**

**0h-6h**

**
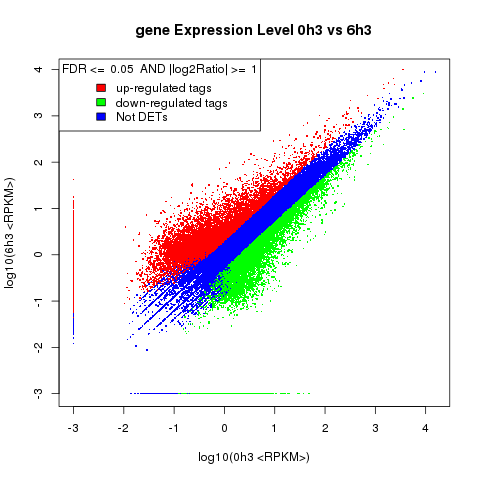

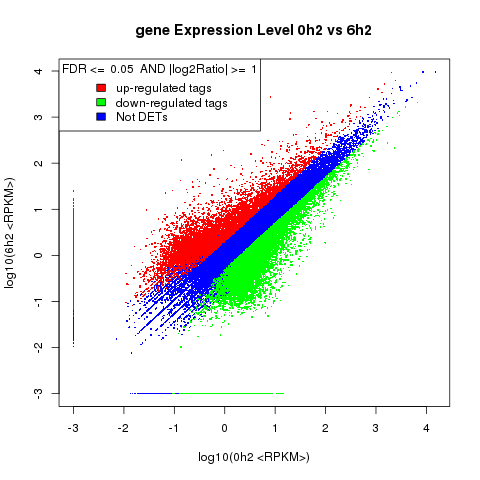

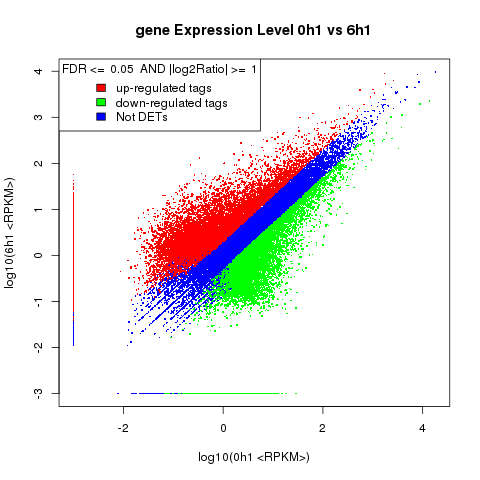
**

**0h-12h**

**
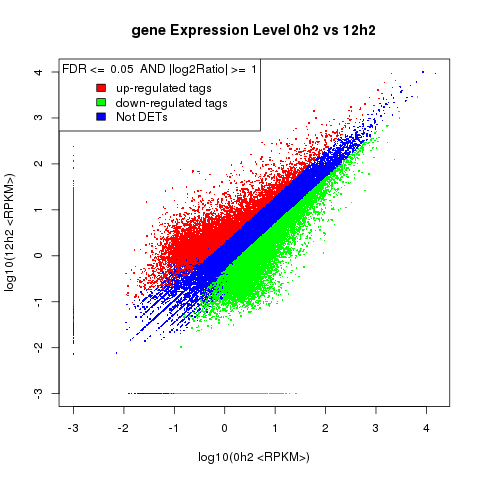

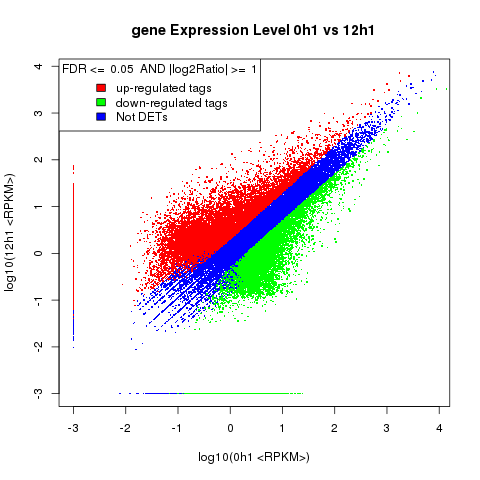

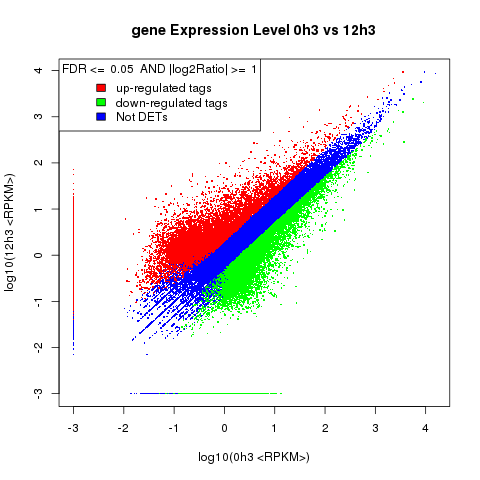
**

**0h-24h**

**
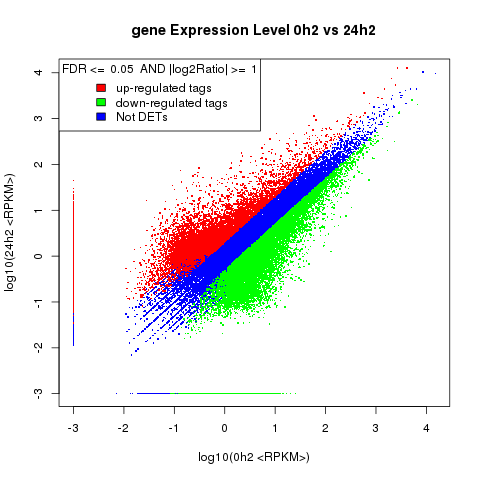

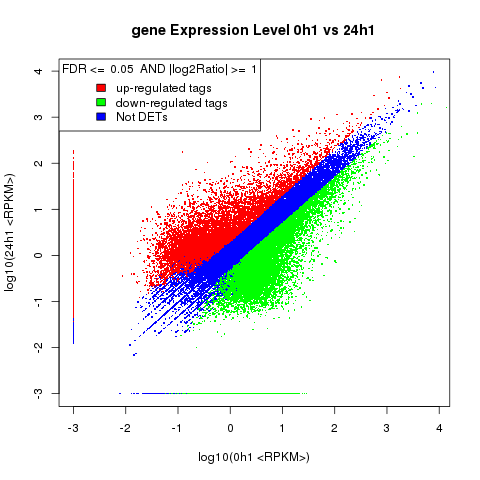

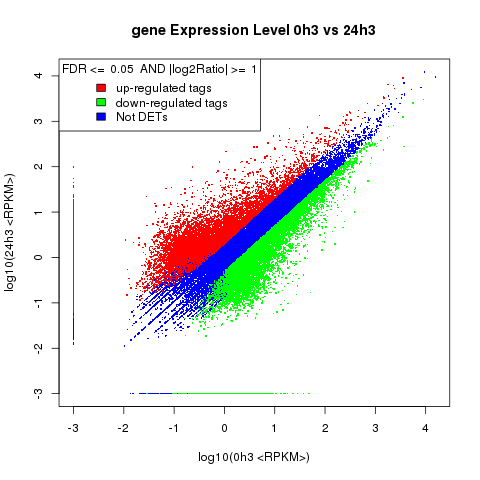
**

**2h-6h**

**
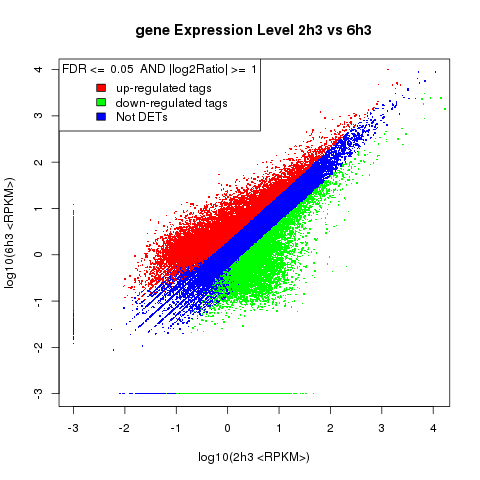

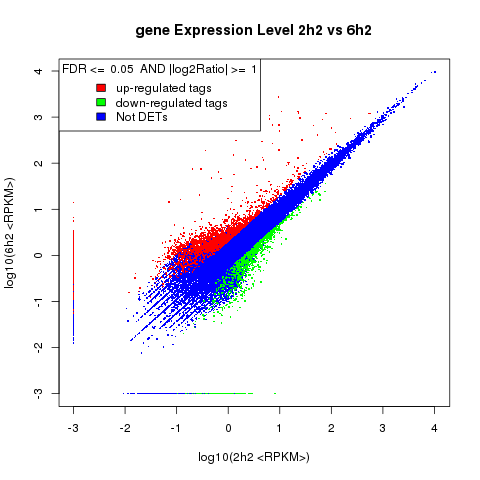

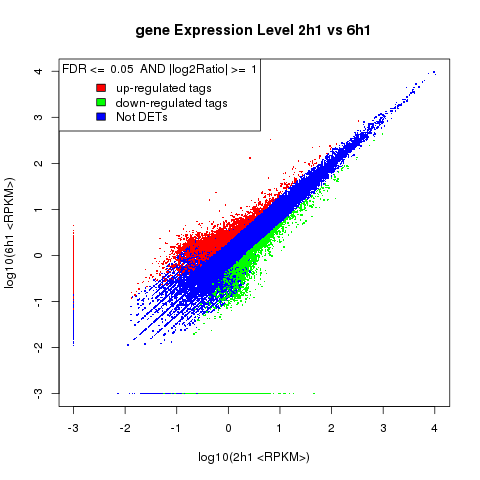
**

**6h-12h**

**
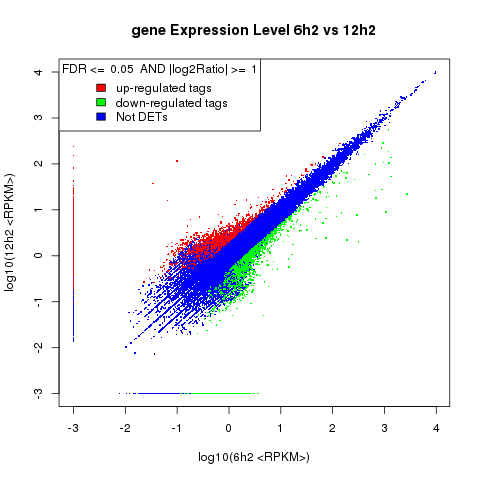

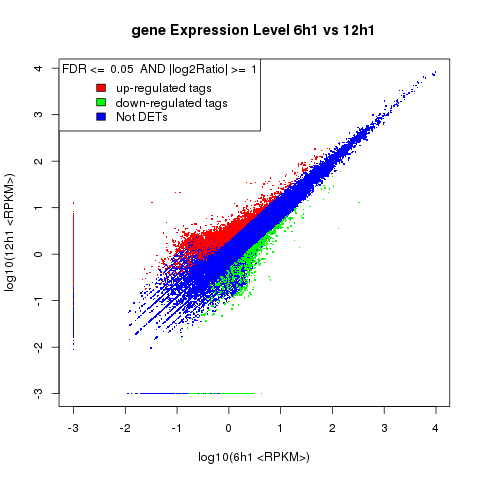

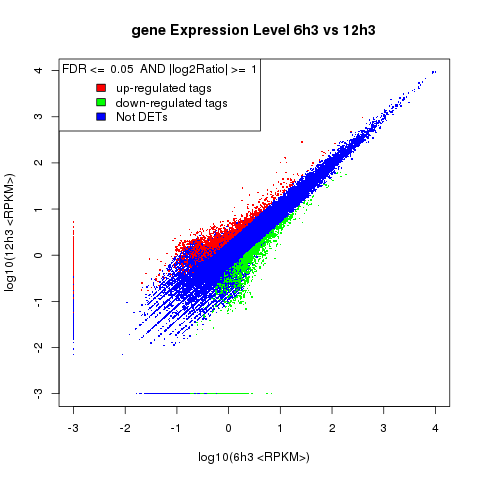
**

**12h-24h**

**
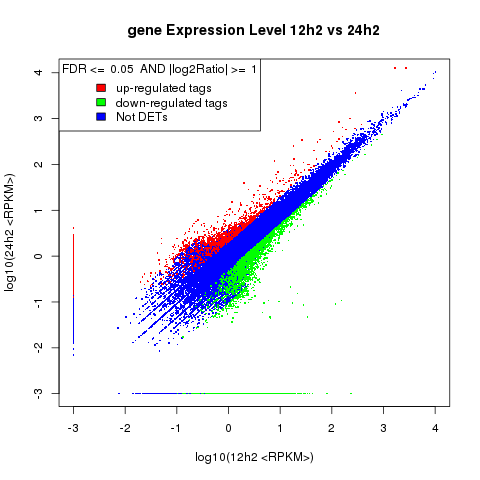

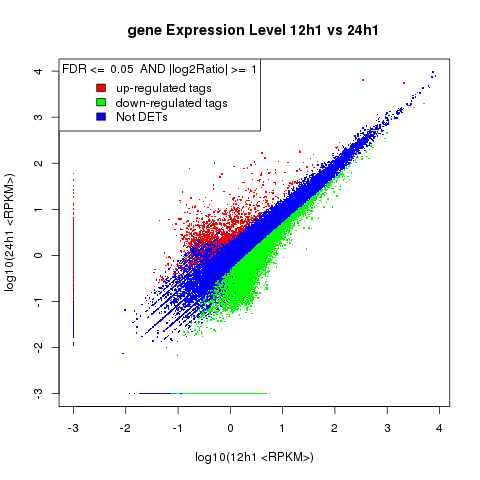

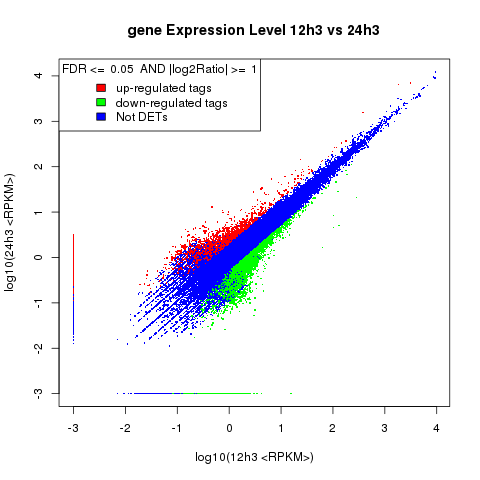
**

Note: The vertical and horizontal coordinates of two samples of the expression of log2 sample1 vs respectively, sample2, red (sample2 relative to the expression of sample1) and green (expression) dots represent gene expression differences (standard for FDR<=0.05, the difference was two times above), blue is no difference.
